# Author Correction: Camrelizumab-based induction chemoimmunotherapy in locally advanced stage hypopharyngeal carcinoma: phase II clinical trial

**DOI:** 10.1038/s41467-024-51057-7

**Published:** 2024-08-07

**Authors:** Hongli Gong, Shu Tian, Hao Ding, Lei Tao, Li Wang, Jie Wang, Tian Wang, Xiaohui Yuan, Yu Heng, Ming Zhang, Yong Shi, Chengzhi Xu, Chunping Wu, Shengzi Wang, Liang Zhou

**Affiliations:** 1 ENT institute and Department of Otorhinolaryngology, Eye & ENT Hospital, Fudan University, Shanghai, 200031 China; 2 Department of Radiation Oncology, Eye & ENT Hospital, Fudan University, Shanghai, 200031 China

**Keywords:** Cancer therapy, Thyroid cancer, Mitosis

Correction to: *Nature Communications* 10.1038/s41467-024-49121-3, published online 19 June 2024

The original version of this Article contained an error in the “Secondary endpoints” section, which incorrectly read “The estimated 2-year OS, PFS, and LPR rates were 83.0% (95% CI, 67.4–91.6%), 77.1% (95% CI, 61.4–87.1%), and 70.0% (95% CI, 51.0–78.8%), 83.0% (95% CI, 67.4–91.6%)”. The correct version removes the “83.0% (95% CI, 67.4–91.6%)” at the end of this sentence. This has been corrected in both the PDF and HTML versions of the Article.

