# Publisher Correction: Author Correction: Camrelizumab-based induction chemoimmunotherapy in locally advanced stage hypopharyngeal carcinoma: phase II clinical trial

**DOI:** 10.1038/s41467-024-52656-0

**Published:** 2024-09-24

**Authors:** Hongli Gong, Shu Tian, Hao Ding, Lei Tao, Li Wang, Jie Wang, Tian Wang, Xiaohui Yuan, Yu Heng, Ming Zhang, Yong Shi, Chengzhi Xu, Chunping Wu, Shengzi Wang, Liang Zhou

**Affiliations:** 1 ENT institute and Department of Otorhinolaryngology, Eye & ENT Hospital, Fudan University, Shanghai, 200031 China; 2 Department of Radiation Oncology, Eye & ENT Hospital, Fudan University, Shanghai, 200031 China

**Keywords:** Head and neck cancer, Head and neck cancer, Cancer immunotherapy

Correction to: *Nature Communications* 10.1038/s41467-024-51057-7, published online 07 August 2024

In the Author Correction, the wrong article title and author list were inadvertently captured.

The title was incorrectly given as ‘Author Correction: dsRNAi-mediated silencing of PIAS2beta specifically kills anaplastic carcinomas by mitotic catastrophe’ but should have been ‘Author Correction: Camrelizumab-based induction chemoimmunotherapy in locally advanced stage hypopharyngeal carcinoma: phase II clinical trial’. The incorrect author list was given and has been updated to match the original article. The original Author Correction article has been corrected.

