# Supplementary material for: Camrelizumab-based induction chemoimmunotherapy in locally advanced stage hypopharyngeal carcinoma: phase II clinical trial
Source: Nat Commun. 2024 Jun 19;15:5251. doi: 10.1038/s41467-024-49121-3 (PMC11187213; doi:10.1038/s41467-024-49121-3)
Supplement: Supplementary file 1 — Supplementary information [file 41467_2024_49121_MOESM1_ESM.pdf]

Supplementary information

Camrelizumab-based induction chemoimmunotherapy in locally advanced stage hypopharyngeal carcinoma: Phase II clinical trial

Hongli Gong[1,*], Shu Tian[2,*], Hao Ding[2], Lei Tao[1], Li Wang[2,] Jie Wang[2,], Tian Wang[2], Xiaohui Yuan[1], Yu Heng[1], Ming Zhang[1], Yong Shi[1], Chengzhi Xu[1], Chunping Wu[1], Shengzi Wang[2,§], Liang Zhou[1,§]

[1] ENT institute and Department of Otorhinolaryngology, Eye & ENT Hospital, Fudan University, Shanghai, 200031, China

[2] Department of Radiation Oncology, Eye & ENT Hospital, Fudan University, Shanghai, 200031, China

* These two authors contributed equally to this work.

[§] Corresponding Authors

Shengzi Wang, Department of Radiation Oncology, Eye & ENT Hospital, Fudan University, Shanghai, 200031, China, Tel: +86-21-64377134, email: shengziwang@fudan.edu.cn

Liang Zhou, Department of Otorhinolaryngology Head and Neck Surgery, Eye & ENT Hospital, Fudan University, Shanghai, 200031, China, Tel: +86-21-64377134, Fax: 86 21 64377151, email: zhoulent@126.com

Supplementary Table 1. Baseline clinical characteristics of 51 patients with LA HSCC.

Supplementary Table 2. The whole treatment (induction chemoimmunotherapy + radioimmunotherapy + immunotherapy maintenance) related adverse events in the intention-to-treat population.

Supplementary Fig. 1. Representative images of laryngoscopy and MRI of two LA HSCC patients showing PR before and after induction chemoimmunotherapy.

Supplementary Fig. 2. RCCEP from patient of No. 27.

Supplementary Fig. 3. Kaplan-Meier survival curves of subgroups.

Supplementary Fig. 4. Peripheral circulating lymphocytes analyses of patients with different therapeutic response before chemoimmunotherapy. Source data are provided as a Source Data file.

Supplementary Note 1. Clinical trial protocol and statistical analysis plan

Tables

Table 1. Baseline clinical characteristics of 51 patients with LA HSCC

	N = 51	%
Smoking		
Smoking	49	96.1
No smoking	2	3.9
Drinking		
Drinking	49	96.1
No Drinking	2	3.9
Clinical stages 7th		
III	11	21.6
IVA	40	78.4
T stages 8th		
T3	28	54.9
T4	23	45.1
N stages 8th		
N0	1	2.0
N1	13	25.5
N2b	11	21.6
N2c	11	21.6
N3	15	29.4
Clinical stages 8th		
III	10	19.6
IVA	26	51.0
IVB	15	29.4

Source data are provided as a Source Data file.

Table 2. The whole treatment (induction chemoimmunotherapy + radioimmunotherapy + immunotherapy maintenance) related adverse events in the intention-to-treat population

Adverse events	Grade 1 - 2	Grade 3	Grade 4
RCCEP	45 (88.2%)	1 (2.0%)	0
Alopecia	51 (100%)	0	0
Dry mouth	44 (86.3%)	0	0
Dysgeusia	40 (78.4%)	0	0
Pharyngeal mucositis	42 (82.4%)	0	0
Dysphagia	43 (84.3%)	0	0
Pneumonia	6 (11.8%)	2 (3.9%)	0
Fever	11 (21.6%)	0	0
Rash	9 (17.6%)	1 (2.0%)	0
Fatigue	37 (72.5%)	0	0
Nausea/vomiting	42 (82.4%)	0	0
Hypothyroidism	7 (13.7%)	0	0
Thyroid stimulating hormone concentration decrease	6 (11.8%)	0	0
Leukopenia	21 (41.2%)	5 (9.8%)	0
Neutropenia	10 (19.6%)	9 (17.6%)	0
Thrombocytopenia	6 (11.8%)	3 (5.9%)	0
Anemia	29 (56.9%)	3 (5.9%)	0
Increased ALT	3 (5.9%)	0	0
Increased AST	3 (5.9%)	0	0
Total bilirubin elevation	4 (7.8%)	0	0
Conjugated bilirubin concentration elevation	3 (5.9%)	0	0
Albuminuria	7 (13.7%)	0	0
Increased creatine kinase	0	0	1 (2.0%)
Increased urea nitrogen	18 (35.3%)	0	0
Arthralgia/Myalgia	0	1(2.0%)	0
Constipation	3 (5.9%)	0	0
Diarrhea	12 (23.5%)	3 (5.9%)	0
Tuberculosis recurrence	0	1 (2.0%)	0
Hand-foot syndrome	3 (5.9%)	0	0
Hypokalemia	2 (3.9%)	0	0
dyspnea	0	1 (2.0%)	0

RCCEP, reactive cutaneous capillary endothelial proliferation; ALT, alanine aminotransferase; AST, aspartate aminotransferase. Source data are provided as a Source Data file.

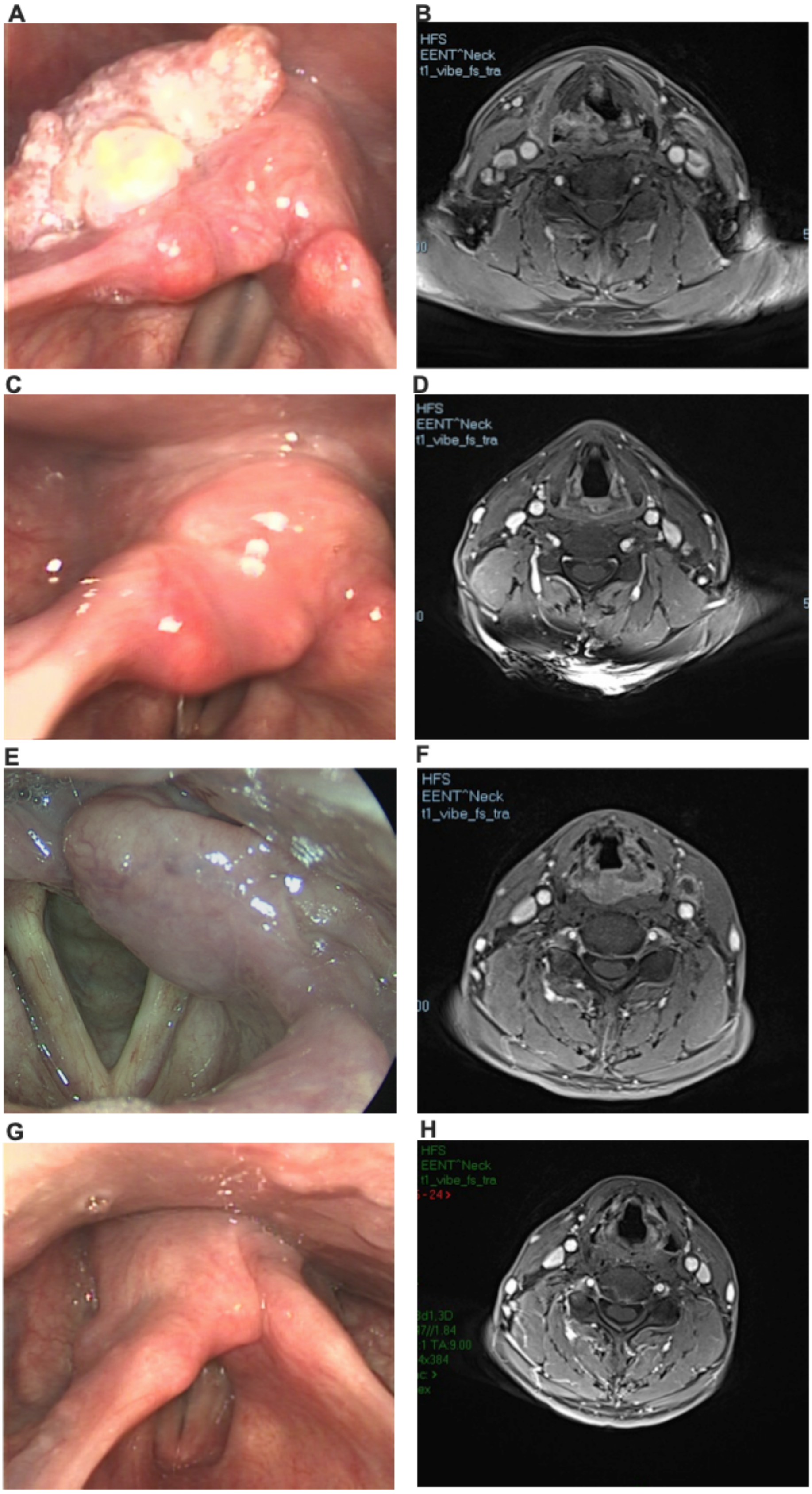

Supplementary Fig. 1. Representative images of laryngoscopy and MRI of two LA HSCC patients showing PR before and after induction chemoimmunotherapy. Images of laryngoscopy

(**A**) and contrast-enhanced MRI (**B**) about primary lesion of patient No. 42 (T4aN2bM0) before induction therapy. Images of laryngoscopy (**C**) and contrast-enhanced MRI (**D**) about primary lesion of patient No. 42 after induction therapy; tumor response showed 40% compared with pre-treatment. Images of laryngoscopy (**E**) and contrast-enhanced MRI (**F**) about primary lesion of patient No. 43 (T3N2bM0) before induction therapy. Images of laryngoscopy (**G**) and contrast-enhanced MRI (**H**) about primary lesion of patient No. 43 after induction therapy; tumor response showed 60% compared with pre-treatment.

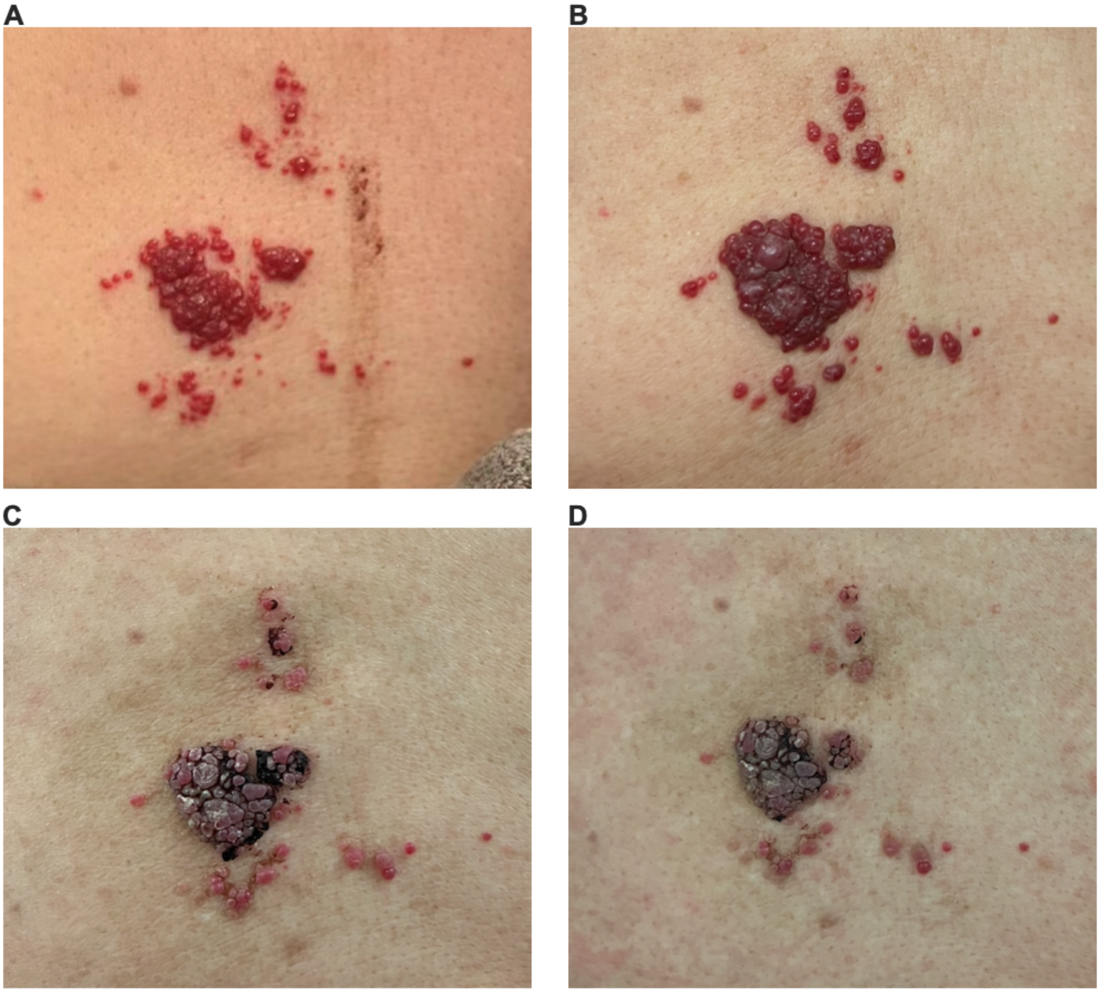

Supplementary Fig. 2. RCCEP from patient of No. 27. (**A**) RCCEP with grade 2 occurred after C2 immunotherapy. (**B**) RCCEP sustained after C4 immunotherapy. (**C**) and (**D**) RCCEP recovering period.

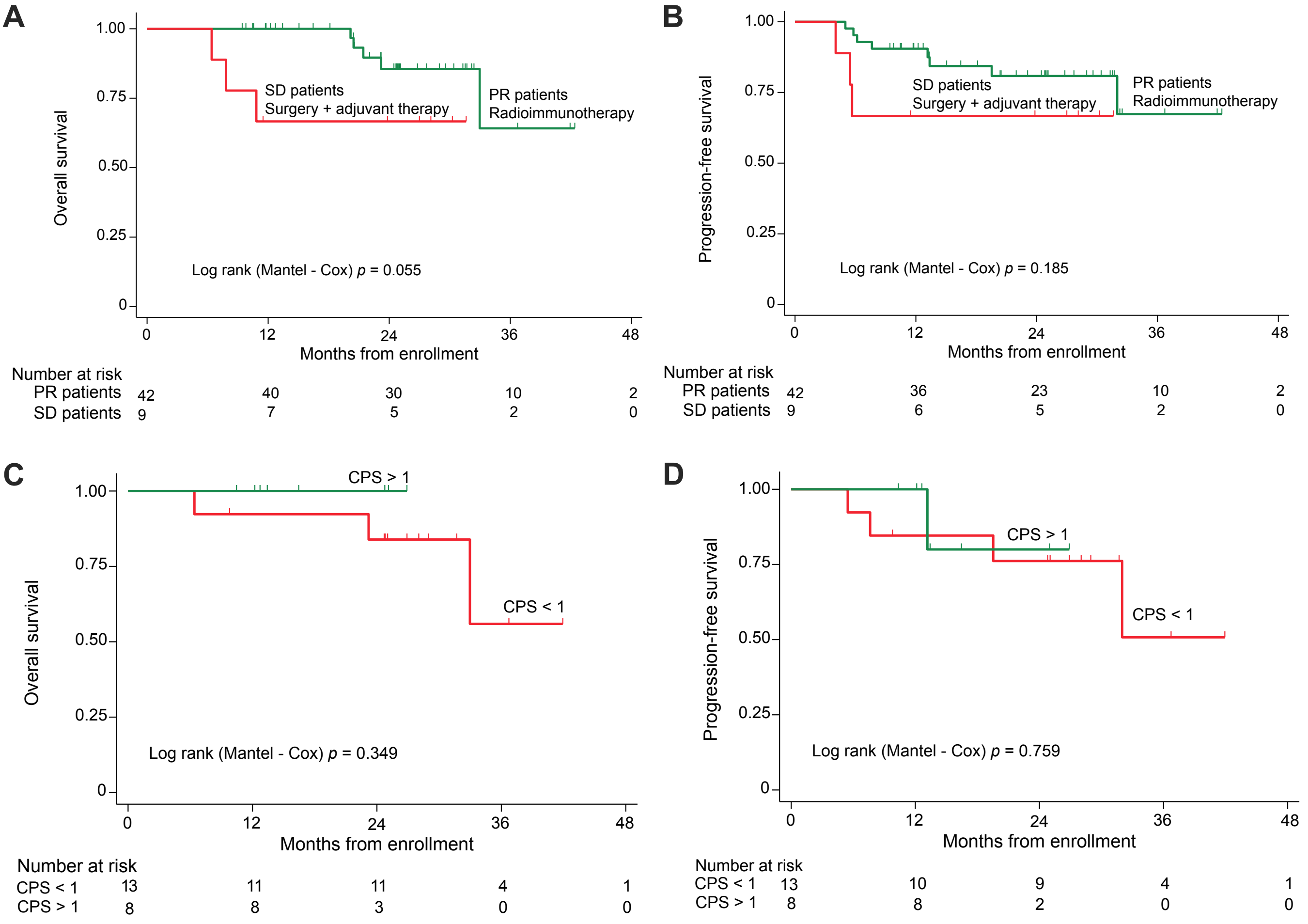

Supplementary Fig. 3. Kaplan-Meier survival curves of subgroups. **(A)** The overall survival curves and the progression-free survival curves **(B)** of patients with PR and SD after induction chemoimmunotherapy. PR patients (n = 42 cases) underwent radioimmunotherapy and SD patients (n = 9 cases) underwent surgery + adjuvant therapy. **(C)** The overall survival curves and the progression-free survival curves **(D)** of patients with CPS <1 (n=13 cases) and CPS> 1 (n=8 cases). Only 21 patients with high quality of immunohistochemistry results were included in this analysis. The estimated 1-year and 2-year OS rate in patients with CPS >1 were both 100% (95% CI, not calculated). The estimated 1-year and 2-year PFS rate in patients with CPS >1 were both 80.0% (95% CI, 20.4% to 96.9%). The estimated 1-year and 2-year OS rate in patients with CPS <1 were 83.6% (95% CI, 48.8% to 95.6%) and 70.8% (95% CI, 32.1% to 90.0%). The estimated 1-year and 2-year PFS rate in patients with CPS <1 were 75.6% (95% CI, 41.8% to 91.4%) and 63.0% (95%CI, 27.2% to 84.9%). This statistical analysis was from log rank test. Source data are provided as a Source Data file.

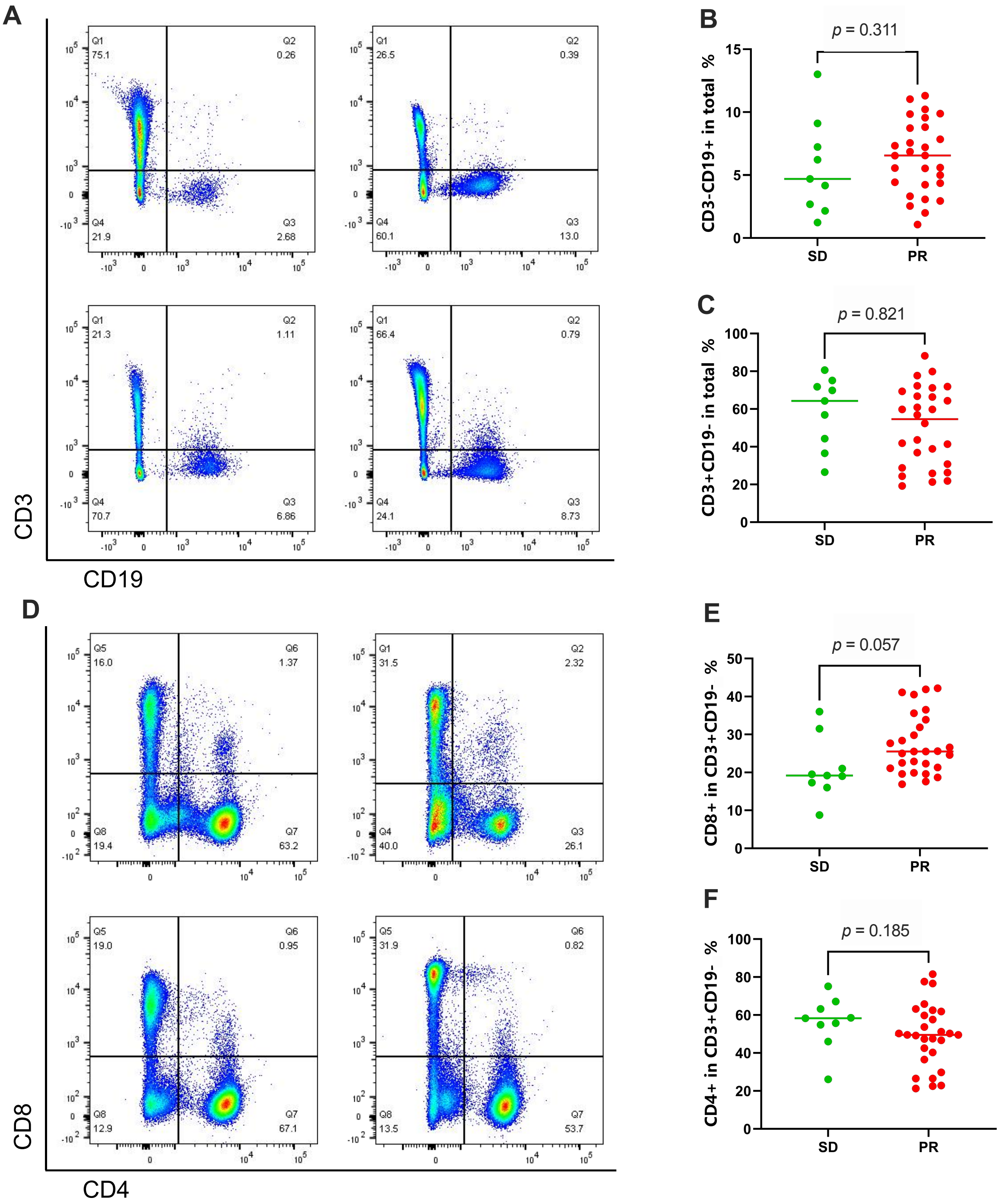

Supplementary Fig. 4. Peripheral circulating lymphocytes analyses of patients with different therapeutic response before chemoimmunotherapy. Percentage of CD3-CD19+ total B lymphocytes (upper column) and CD3+CD19- total T lymphocytes (lower column) between patients with PR and patients with SD (**A**), and the corresponding statistical analysis graphs of B cells (**B**) and T cells (**C**) were shown (PR patients n = 9 cases versus SD patients n = 28 cases). Percentage of CD8+ (upper column) and CD4+ (lower column) T lymphocytes in total

CD3+CD19- T cells between patients with PR and patients with SD (**D**), and the corresponding statistical analysis graphs of CD8+ cells (**E**) and CD4+ cells (**F**) were shown (PR patients n = 9 cases versus SD patients n = 28 cases). The statistical significance was tested using a Mann–Whitney test (two-sided), and the line represent median. Source data are provided as a Source Data file.

Supplementary Note 1

Clinical trial protocol and statistical analysis plan

Drug name:	Camrelizumab
Code name:	SHR-1210
Version:	1.1(2019-07-26)
Date:	2019-07-26
ClinicalTrials.gov ID:	NCT04156698
Study phase	II

Principal Investigator: Liang Zhou and Shengzi Wang

This document contains confidential information. If any actual or suspected violation of this obligation is discovered, Liang Zhou and Shengzi Wang must be notified immediately.

This trial is registered with ClinicalTrials.gov.

The interim analysis was authorized by the Committee of Data and Safety Monitoring Board at Eye & ENT Hospital, and the authorization was obtained.

This file contains the following items:

1. Original protocol, final protocol, summary of changes.

2. Original statistical analysis plan, final statistical analysis plan, summary of changes.

Table of contents

Abstract (summarized from final protocol) 4
Original protocol (Version 1.1(2019-07-26)) 8
Background 8
Study design 12
Arm and interventions 12
Treatment drugs 14
Treatment plan 15
Eligibility criteria 15
Outcome measures 18
System evaluation and head and neck cancer examination 19
Pathology and biomarker examination 19
Imaging examination 20
Safety profile evaluation 20
Quality of life score 20
Peripheral blood and tumor tissue samples 20
Toxic side effects, treatment discontinue, and dosage adjustment 21
Statistical analysis 21
Adverse event 22
Follow-up 24
The relationship between adverse events (AE) and treatment evaluation 24
Patient withdrew from the trial 24
Ethics 25
Clinical trial management and quality control 25
Appendix 27
Final protocol (Version 1.2(2021-08-08)) 31
Background 31
Study design 35
Arm and interventions 35
Treatment drugs 37
Treatment plan 38
Eligibility criteria 38
Outcome Measures 41
System evaluation and head and neck cancer examination 42
Pathological examination 42
Imaging examination 43
Safety profile evaluation 43
Quality of life score 43
Peripheral blood and tumor tissue samples 43
Toxic side effects, treatment discontinue, and dosage adjustment 44
Statistical analysis 44
Adverse event 45
Follow-up 47
The relationship between adverse events (AE) and treatment evaluation 47

Patient withdrew from the trial 47
Ethics 48
Clinical trial management and quality control 48
Appendix 50
Summary of changes – Protocol 54
Original statistical analysis plan (version 1.1(2019-07-26)) 55
Study design 55
Primary outcome measure 55
Secondary outcome measures (time frame: 3 years) 55
Baseline clinical characteristics 55
Adverse events 57
Oncologic outcomes 57
Statistical analyses overview 58
Sample size calculation 59
Baseline clinical characteristics analyses 59
Adverse events evaluation 60
Figures 61
Subgroups analyses 63
Final statistical analysis plan (Version 1.2(2021-08-08)) 68
Study design 68
Primary outcome measure (time frame: 9 weeks) 68
Secondary outcome measures (time frame: 3 years) 68
Baseline clinical characteristics 68
Adverse events 70
Oncologic outcomes 70
Statistical analyses overview 71
Sample size calculation 72
Baseline clinical characteristics analyses 72
Adverse events evaluation 73
Figures 74
Subgroups analyses 76
Summary of changes – statistical analysis plan 81

Abstract (summarized from final protocol)

<table>
<tr><td colspan="2">Official title</td><td>A Phase II, Single-center, Open-label, Single-arm Study of Induction Chemotherapy Combined With Immunotherapy for Locally Advanced Hypopharyngeal Carcinoma</td></tr>
<tr><td colspan="2">Brief title</td><td>Induction Chemotherapy Combined With Immunotherapy for Locally Advanced Hypopharyngeal Carcinoma</td></tr>
<tr><td colspan="2">Version</td><td>1.2(2021-08-08)</td></tr>
<tr><td colspan="2">Sponsors</td><td>Department of Otorhinolaryngology Head and Neck surgery and Department Radiation Oncology, Eye & ENT Hospital, Fudan University</td></tr>
<tr><td colspan="2">Study design</td><td>Single-center, multiple discipline, open-label, prospective, exploratory single-arm study</td></tr>
<tr><td colspan="2">Enrollment</td><td>Locally advanced stage hypopharyngeal carcinoma patients who have to undergo total laryngectomy as an initial therapy</td></tr>
<tr><td rowspan="2">Outcome measures</td><td>Primary outcome measure</td><td>Overall Response Rate (ORR) that proportion of patients with complete or partial response (CR or PR) in tumor burden as defined by Response Evaluation Criteria in Solid Tumors (RECIST) version 1.1.</td></tr>
<tr><td>Secondary outcome measure</td><td>3 years - laryngeal preservation rate (LPR)
3 years - progression free survival (PFS)
3 years - metastasis free survival (MFS)
3 years - overall survival (OS)</td></tr>
<tr><td colspan="2">Number of enrolled patients</td><td>51 patients</td></tr>
<tr><td colspan="2">Principal investigators</td><td>Prof. Liang Zhou and Prof. Shengzi Wang</td></tr>
<tr><td colspan="2">Institution</td><td>Eye & ENT Hospital, Fudan University</td></tr>
<tr><td colspan="2">Eligibility</td><td>Inclusion criteria:
1. Patients have histologically confirmed hypopharyngeal squamous cell carcinoma and require total laryngectomy, including the piriform fossa, postcricoid region, and posterior pharyngeal wall with TNM stage cT3-4aN0-2M0 (AICC 7th).
2. Able to understand and willing to sign a written informed consent document.
3. Age ≥ 18 and ≤ 70 years.
4. Male or female.
5. Performance status of ECOG 0-2.
6. Expected lifetime > 6 months.
7. Normal blood test, hepatic and renal functions. Normal hearing. Blood test: WBC ≥ 4.0×10^9/L, ANC ≥ 2.0×10^9/L, PLT ≥100×10^9/L, HGB ≥ 100g/ L. Hepatic function: ALT and AST< upper limit of normal. Kidney function: serum creatinine < upper limit of normal</td></tr>
</table>

	value, and creatinine clearance rate ≥ 60 ml/min Cockcroft-Gault formula. Cardiac ultrasonography left ventricular ejection fraction > 50%.<br>8. No prior allergic reaction to biological agents and/or ingredient in the drug.<br>9. No drug abuse.<br>10. Good compliance.<br>11. No systemic diseases (such as other tumors, severe heart, lung and central nervous system diseases, etc.).<br>12. Negative pregnancy test (for female patients with fertility).<br>13. Male patients with fertility and female patients with fertility and pregnancy risk must agree to use contraceptive methods throughout the study period, and continued until at least 6 months after the last dose of cisplatin and 30 days after the last dose of PD-1 antibody/placebo (whichever occurs later). Female patients do not have fertility (ie meet at least one of the following criteria): underwent hysterectomy and/or bilateral oophorectomy with archival records, medically confirmed ovarian function decline, in postmenopausal state. The Postmenopausal is defined as: at least 12 months of continuous menstruation without other pathological or physiological reasons, and the status confirmed by serum follicle stimulating hormone (FSH) levels is consistent with postmenopausal status.<br><br>Exclusion criteria:<br>1. Patients with cervical lymph node cN3 (AJCC 7th)；<br>2. Have a history of other cancers in the past five years, patients with radical or untreated prostate cancer (Gleason score ≤ 6), or complete treatment of breast ductal carcinoma in situ; except for cured skin basal cell carcinoma or squamous cell skin cancer.<br>3. Patients with target lesions who have received radiation therapy or surgery (except biopsy).<br>4. Patients who have previously used chemotherapy, immunotherapy, or biological targeted therapy for primary tumors.<br>5. Patients who have participated in other clinical trials within 4 weeks before the test.<br>6. Any of the following conditions within 6 months before random grouping: myocardial infarction, severe/unstable angina, coronary artery/peripheral artery bypass graft, symptomatic congestive heart failure, cerebrovascular accident, patients with transient ischemic attack or symptomatic pulmonary embolism.<br>7. Patients with hypertension who cannot control well through single antihypertensive medication (systolic blood pressure >140 mmHg, diastolic blood pressure > 90 mmHg).

	8. Patients with grade I or above coronary heart disease, arrhythmia (including men with a QTc interval > 450 ms, women > 470 ms), and cardiac insufficiency.<br>9. Urinary protein was greater than ++ and 24-hour urinary protein quantification > 1.0 g.<br>10. Many factors that affect oral medications (such as inability to swallow, nausea, vomiting, chronic diarrhea, and intestinal obstruction).<br>11. Patients with abnormal coagulation function (INR>1.5, APTT>1.5 ULN), with bleeding tendency.<br>12. Patients with a history of psychotropic substance abuse that is active or has a mental disorder.<br>13. Patients who required systemic treatment with corticosteroids (>10 mg prednisone equivalent daily) or other immunosuppressive agents within 2 weeks prior to the first use of the study drug.<br>14. Patients with a history of severe allergies or allergies; patients with active autoimmune diseases that may worsen when receiving immunostimulants; patients with type 1 diabetes, vitiligo, psoriasis, or hypothyroidism or hyperthyroidism who do not require immunosuppressive therapy are eligible to participate in the study.<br>15. Patients who have previously been diagnosed with immunodeficiency or known human immunodeficiency virus (HIV) or acquired immunodeficiency syndrome (AIDS)-related diseases. Hepatitis B virus (HBV) or hepatitis C virus (HCV) infection, HBV surface antigen is positive at screening, or patients with positive HCV RNA (ribonucleic acid) when positive for anti-HCV antibody screening test.<br>16. Vaccination within 4 weeks prior to randomization, except for inactivated vaccines.<br>17. Pregnant or lactating women who are in the reproductive period but have not taken effective contraceptive measures.<br>18. The investigator believes that it is inappropriate for individuals to participate in the trial: having, for example, severe acute or chronic medical conditions (including immune colitis, inflammatory bowel disease, non-infectious pneumonia, pulmonary fibrosis) or mental illness (including recent time (within the past year) or active suicidal ideation or behavior).<br>19. With a past history of tuberculosis or antituberculosis treatment.
Study status	Actual study start date: May 21, 2020<br>Primary completion date: April 15, 2023<br>Study completion date: January 16, 2026
Arms and interventions	Camrelizumab (PD-1 inhibitor) group<br>Induction chemotherapy combined with immunotherapy (TPF + camrelizumab), q3w, 3 cycles in total: docetaxel (domestic) 75 mg/m^2 i.v.

	d1, cisplatin 25 mg/m^2 i.v. d1-3, capecitabine 800 mg/m^2 po bid d1-d14, camrelizumab 200 mg i.v. d1;<br><br>Radical radiotherapy followed by induction chemoimmunotherapy<br>Radiotherapy: Using intensity-modulated radiation therapy (IMRT). Primary site: GTV dose 66 (2.2Gy / fraction)-70 Gy (2Gy / fraction); CTV 1.6-1.9 Gy / fraction. Cervical lymph nodes: Radiotherapy plan is the same as the radiotherapy plan of primary site; concurrent immunotherapy: camrelizumab 200 mg i.v. d1, d22;<br><br>Maintenance period<br>After completing concurrent radiotherapy combined with immunotherapy, camrelizumab 200 mg q3w will be given every three weeks up to 12 months (calculated from the time of the first dose of PD-1 immunotherapy).
Safety profile	Adverse events (AE) and laboratory safety will be monitored before each treatment initiation and graded using the National Cancer Institute Common Terminology Criteria for Adverse Events (version 5.0).

Original protocol (Version 1.1(2019-07-26))

Background

Head and neck cancer is the sixth most common malignant tumor, and hypopharyngeal cancer is one common and aggressive type of head and neck cancer, displaying inferior prognosis generally[1]. Approximately 80% of patients with hypopharyngeal cancer have locally advanced (stage III and IV) lesions at the time of diagnose, and the 5-year overall survival rate for surgical treatment of locally advanced hypopharyngeal cancer is 30% in China[2]. The current treatments includes a combination of surgery, radiation therapy, chemotherapy, and immunotherapy. Although the surgical approach and radio/chemotherapy regimen continuing reform, the survival rate of patients has not been significantly improved[2]. Additionally, 30% to 40% of patients die due to difficult treatment of recurrence or metastasis despite undergoing radical therapy[1,2]. Therefore, effective clinical treatment plans to preserve laryngeal function and improve survival outcomes have become a challenge for head and neck surgeons and oncologists.

Currently, comprehensive treatment with surgery plus postoperative radio/chemotherapy for advanced head and neck cancer is the main therapy regimen in China. Most advanced laryngeal and hypopharyngeal cancer patients lose their normal pronunciation and respiratory function after total laryngectomy, which brings serious functional impairment and psychological problems to them[3]. In recent years, there have been significant changes in the treatment plans for advanced laryngeal and hypopharyngeal cancer[3]. The important change is derived from the representative clinical trial reported by the Veterans Affairs study, which found that cisplatin+5-fluorouracil induced chemotherapy (PF) plus radiotherapy for patients with advanced laryngeal cancer, without sacrificing the survival rate, to a greater extent preserve the laryngeal function, and improve the treatment of advanced laryngeal cancer to non-surgical treatment[4]. Besides, EORTC 24891 study finds the improved regimen of treatment for hypopharyngeal cancer [5]. Patients receive PF induced chemotherapy, and those who responded effectively receive radical radiotherapy; the rest patients receive surgery and postoperative radiotherapy. There is

no difference in treatment failure rates between these two groups in terms of local, cervical lymph nodes, and secondary primary cancer, but the induction chemotherapy group have a lower rate of distant metastasis and a longer median survival time compared with surgery and postoperative radiotherapy[5]. However, the study by PARADIGM demonstrates that the clinical efficacy of TPF induced chemotherapy combined with radiochemotherapy and concurrent radiochemotherapy in the treatment of advanced head and neck cancer is similar; there is no significant difference in overall survival between these two groups (73% vs 78%)[6]. Induction chemotherapy with TPF, an evidence-based option for advanced HNSCC, establishes a favorable response and milder toxicity profile and improves survival outcomes and function preservation from pooled data analyses (TAX 323/EORTC 24971, TAX 324, GORTEC 2000-01, and TTCC 2002) [7]. Due to different national conditions, these non-surgical treatment plans for larynx preservation have not been widely accepted by many hospitals in the treatment of head and neck cancer in China, and further study is required to confirm the differences among these therapy regimens.

Recently, immune checkpoint inhibitors such as humanized anti-programmed death receptor 1 (PD-1) antibodies have presented efficacy and safety in unresectable head and neck cancer. Patients with recurrent or metastatic disease after platinum chemotherapy treated with nivolumab monoclonal antibody have prolonged overall survival over standard and single-agent therapy treatment.[8] Pembrolizumab monotherapy is well tolerated, and shows clinically substantial antitumor activity in patients with recurrent or metastatic head and neck cancer, with characteristics of prolonging overall survival and favorable safety profile than standard care therapy[9]. Camrelizumab (SHR-1210), another PD-1 monoclonal antibody, is a well-tolerated and appropriate option for patients with recurrent or metastatic nasopharyngeal carcinoma[10]. Camrelizumab monotherapy, gemcitabine, and cisplatin present an acceptable toxicity profile and encourage antitumor activity for patients with treatment-naive recurrent or metastatic nasopharyngeal carcinoma[10]. The discussion of the best practices for head and neck cancer remains ongoing, and the efficacy and safety profile of camrelizumab combined with TPF in head and neck cancer are still unknown.

The purpose of this study is to evaluate the oncological outcome, laryngeal preservation,

and safety profile of camrelizumab in combination with a modified TPF (docetaxel, cisplatin, and capecitabine) regimen as induction therapy for locally advanced hypopharyngeal cancer patients who have previously untreated history and required total laryngectomy.

Reference

1. Chen W, Zheng R, Baade PD, et al. Cancer statistics in China, 2015. CA Cancer J Clin 2016;66(2):115-32.

2. Expert consensus on surgery and comprehensive treatment of hypopharyngeal carcinoma. Zhonghua Er Bi Yan Hou Tou Jing Wai Ke Za Zhi. 2017. 52(1):16-24. doi: 10.3760/cma.j.issn.1673-0860.2017.01.004.

3. Steuer CE, El-Deiry M, Parks JR, Higgins KA, Saba NF. An update on larynx cancer. CA Cancer J Clin 2017;67(1):31-50.

4. Department of Veterans Affairs Laryngeal Cancer Study G, Wolf GT, Fisher SG, et al. Induction chemotherapy plus radiation compared with surgery plus radiation in patients with advanced laryngeal cancer. N Engl J Med 1991;324(24):1685-90.

5. Lefebvre JL, Chevalier D, Luboinski B, Kirkpatrick A, Collette L, Sahmoud T. Larynx preservation in pyriform sinus cancer: preliminary results of a European Organization for Research and Treatment of Cancer phase III trial. EORTC Head and Neck Cancer Cooperative Group. J Natl Cancer Inst 1996;88(13):890-9.

6. Haddad R, O'Neill A, Rabinowits G, et al. Induction chemotherapy followed by concurrent chemoradiotherapy (sequential chemoradiotherapy) versus concurrent chemoradiotherapy alone in locally advanced head and neck cancer (PARADIGM): a randomised phase 3 trial. Lancet Oncol 2013;14(3):257-64.

7. Haddad RI, Posner M, Hitt R, et al. Induction chemotherapy in locally advanced squamous cell carcinoma of the head and neck: role, controversy, and future directions. Ann Oncol 2018;29(5):1130-1140.

8. Ferris RL, Blumenschein G, Jr., Fayette J, et al. Nivolumab for Recurrent Squamous-Cell Carcinoma of the Head and Neck. N Engl J Med 2016;375(19):1856-1867.

9. Seiwert TY, Burtness B, Mehra R, et al. Safety and clinical activity of pembrolizumab for

treatment of recurrent or metastatic squamous cell carcinoma of the head and neck (KEYNOTE-012): an open-label, multicentre, phase 1b trial. Lancet Oncol 2016;17(7):956-965.

10. Fang W, Yang Y, Ma Y, et al. Camrelizumab (SHR-1210) alone or in combination with gemcitabine plus cisplatin for nasopharyngeal carcinoma: results from two single-arm, phase 1 trials. Lancet Oncol 2018;19(10):1338-1350.

Study design

This is an open-label, single-arm, phase II, prospective, multidisciplinary and single-center study, and is sponsored and performed by Department of Otorhinolaryngology Head and Neck surgery and Department Radiation Oncology, Eye & ENT Hospital, Fudan University, Shanghai.

The purpose of this study is to assess antitumor activity and safety profile of camrelizumab (PD-1 inhibitor) plus modified TPF (docetaxel, cisplatin, and capecitabine) of induction therapy for patients with locally advanced hypopharyngeal squamous cell carcinoma (LA HSCC). Enrolled patients involved TNM stage of T3-4aN0-2M0 (AJCC 7th), with types of the pyriform sinus, postcricoid region, and posterior hypopharyngeal wall.

The PD-1 monoclonal antibody is camrelizumab (SHR-1210) from Hengrui Medicine Company, Jiangsu province, China.

Arm and interventions

Single arm with camrelizumab group (induction chemoimmunotherapy)

Induction chemotherapy combined with immunotherapy (TPF + camrelizumab), q3w, three cycles:

TPF chemotherapy

Docetaxel (domestic) 75 mg/m^2 i.v. d1

Cisplatin 25 mg/m^2 i.v. d1-3

Capecitabine 800 mg/m^2 po bid d1-d14

Immunotherapy

Camrelizumab 200 mg i.v. d1

Radical radiotherapy followed by induction chemoimmunotherapy:

Radiotherapy

Intensity-modulated radiation therapy (IMRT). Primary site: GTV dose 66 (2.2 Gy/fraction) - 70 Gy (2 Gy/fraction); CTV 1.6 - 1.9 Gy/fraction. Cervical lymph nodes: radiotherapy plan is the same as the radiotherapy plan of primary lesion.

Concurrent immunotherapy

Camrelizumab 200 mg i.v. d1, d22

Maintenance period

After completing concurrent radiotherapy combined with immunotherapy, camrelizumab 200 mg q3w and apatinib 250 mg qd will be given up to 12 months (calculated from the time of the first dose of PD-1 immunotherapy). The dose of camrelizumab could not be modified.

Surgical treatment

When patients have stable disease (SD) or progress disease (PD) that are evaluated after induction chemoimmunotherapy, they will be assigned to surgical treatment followed by adjuvant (chemo)radiotherapy. If there is further evidence of uncontrolled or progression disease, camrelizumab will be discontinued. Treatment could be discontinued at the patient's or clinician's discretion. Salvage surgery will be conducted if patients have uncontrolled or progressive disease or locoregional recurrence.

Adjuvant therapy

Patients with high-risk factors after total laryngectomy are treated with adjuvant radiotherapy or chemoradiotherapy

Adjuvant chemoradiotherapy

Patients with extranodal extension with or without positive surgical margins receive adjuvant chemoradiotherapy postoperatively.

Adjuvant radiotherapy

Patients with multiple positive nodes (without extranodal extension), perineural invasion, vascular invasion, lymphatic invasion, pT3 or pT4 primary, pN2 or pN3 will receive adjuvant radiotherapy postoperative treatment.

Intensity-modulated radiation therapy (IMRT).

Primary site: GTV dose 66 - 70 Gy: chemoradiotherapy plan, 1.8 - 2 Gy/fraction, radiotherapy plan, 1.8 – 2.2 Gy/fraction.

Cervical lymph nodes: radiotherapy plan is the same as the radiotherapy plan of primary site.

Chemotherapy

Cisplatin 25 mg/m^2 i.v. d1-3, d22-24

Treatment drugs

Docetaxel is a chemotherapy drug

Cisplatin is a chemotherapy drug.

Capecitabine is a chemotherapy drug.

Camrelizumab (SHR-1210) is a humanized anti-PD1 IgG4 monoclonal antibody (Jiangsu Hengrui). Hengrui Medicine Co. partially donated the study drug.

Treatment plan

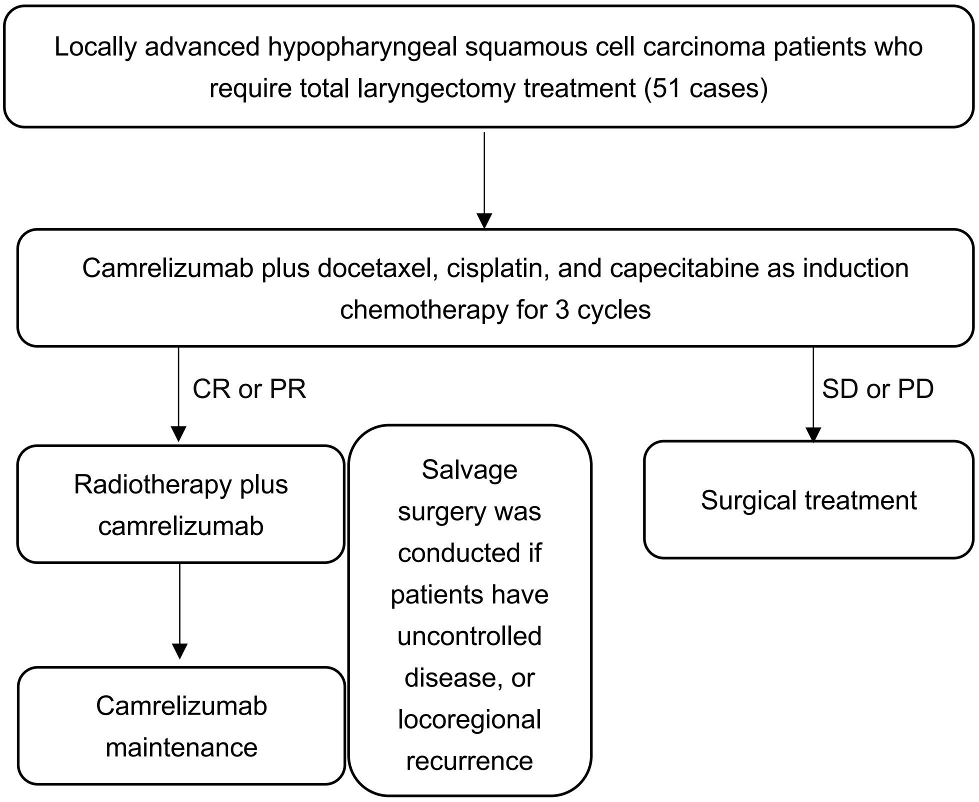

Eligibility criteria

Inclusion criteria:

1. Patients have histologically confirmed hypopharyngeal squamous cell carcinoma and require total laryngectomy, including the piriform fossa, postcricoid region, and posterior pharyngeal wall with TNM stage cT3-4aN1-2M0 (AJCC 7th).
2. Able to understand and willing to sign a written informed consent document.
3. Age ≥ 18 and ≤ 70 years.
4. Male or female.
5. No distant metastasis, M0.
6. Performance status of ECOG 0-2.
7. Expected lifetime > 6 months.
8. Normal blood test, hepatic and renal functions. Normal hearing. Blood test: WBC ≥

4.0×10^9/L, ANC ≥ 2.0×10^9/L, PLT ≥100×10^9/L, HGB ≥ 100g/ L. Hepatic function: ALT and AST< upper limit of normal. Kidney function: serum creatinine < upper limit of normal value, and creatinine clearance rate ≥ 60 ml/min Cockcroft-Gault formula. Cardiac ultrasonography left ventricular ejection fraction > 50%.

9. No prior allergic reaction to apatinib/biological agents and/or ingredient in the drug.
10. No drug abuse.
11. Good compliance.
12. No systemic diseases (such as other tumors, severe heart, lung and central nervous system diseases, etc.).
13. Negative pregnancy test (for female patients with fertility).
14. Male patients with fertility and female patients with fertility and pregnancy risk must agree to use contraceptive methods throughout the study period, and continued until at least 6 months after the last dose of cisplatin and 30 days after the last dose of PD-1 antibody/placebo (whichever occurs later). Female patients do not have fertility (ie meet at least one of the following criteria): underwent hysterectomy and/or bilateral oophorectomy with archival records, medically confirmed ovarian function decline, in postmenopausal state. The Postmenopausal is defined as: at least 12 months of continuous menstruation without other pathological or physiological reasons, and the status confirmed by serum follicle stimulating hormone (FSH) levels is consistent with postmenopausal status.

Exclusion criteria:

1. Patients with cervical lymph node cN3 (AJCC 7th)；
2. Have a history of other cancers in the past five years, patients with radical or untreated prostate cancer (Gleason score ≤ 6), or complete treatment of breast ductal carcinoma in situ, except for cured skin basal cell carcinoma or squamous cell skin cancer.
3. Patients with target lesions who have received radiation therapy or surgery (except biopsy).
4. Patients who have previously used chemotherapy, immunotherapy, or biological targeted therapy for primary tumors.

5. Patients who have participated in other clinical trials within 4 weeks before the test.
6. Any of the following conditions within 6 months before random grouping: myocardial infarction, severe/unstable angina, coronary artery/peripheral artery bypass graft, symptomatic congestive heart failure, cerebrovascular accident, patients with transient ischemic attack or symptomatic pulmonary embolism.
7. Patients with hypertension who cannot control well through single antihypertensive medication (systolic blood pressure >140 mmHg, diastolic blood pressure > 90 mmHg).
8. Patients with grade I or above coronary heart disease, arrhythmia (including men with a QTc interval > 450 ms, women > 470 ms), and cardiac insufficiency.
9. Urinary protein was greater than ++ and 24-hour urinary protein quantification > 1.0 g.
10. Many factors that affect oral medications (such as inability to swallow, nausea, vomiting, chronic diarrhea, and intestinal obstruction).
11. Patients with abnormal coagulation function (INR>1.5, APTT>1.5 ULN), with bleeding tendency.
12. Patients with a history of psychotropic substance abuse that is active or has a mental disorder.
13. Patients who required systemic treatment with corticosteroids (>10 mg prednisone equivalent daily) or other immunosuppressive agents within 2 weeks prior to the first use of the study drug.
14. Patients with a history of severe allergies or allergies; patients with active autoimmune diseases that may worsen when receiving immunostimulants; patients with type 1 diabetes, vitiligo, psoriasis, or hypothyroidism or hyperthyroidism who do not require immunosuppressive therapy are eligible to participate in the study.
15. Patients who have previously been diagnosed with immunodeficiency or known human immunodeficiency virus (HIV) or acquired immunodeficiency syndrome (AIDS)-related diseases. Hepatitis B virus (HBV) or hepatitis C virus (HCV) infection, HBV surface antigen is positive at screening, or patients with positive HCV RNA (ribonucleic acid) when positive for anti-HCV antibody screening test.
16. Vaccination within 4 weeks prior to randomization, except for inactivated vaccines.

17. Pregnant or lactating women who are in the reproductive period but have not taken effective contraceptive measures.
18. The investigator believes that it is inappropriate for individuals to participate in the trial: having, for example, severe acute or chronic medical conditions (including immune colitis, inflammatory bowel disease, non-infectious pneumonia, pulmonary fibrosis) or mental illness (including recent time (within the past year) or active suicidal ideation or behavior).

Outcome measures

Primary outcome measure (time frame: 9 weeks):

Overall response rate (ORR)

ORR is defined as the proportion of patients with best response of complete or partial response (CR or PR) in tumor burden of primary lesion as defined by RECIST 1.1.

The tumor volume system is also used to evaluate the response of three dimensions of the primary lesion during treatment, and PR is defined as volume regression of 70% compared with baseline.

Secondary outcome measure (time frame: 3 years):

Larynx preservation rate (LPR)

LPR is defined as the time from initial treatment to a total laryngectomy, dysfunction of the larynx, or death.

Progression free survival (PFS)

PFS is defined as the time from initial treatment to the first documented disease progression or death due to any cause, whichever occurred first.

Metastasis free survival (MFS)

MFS is defined as the time from initial treatment to distant metastasis.

Overall survival (OS)

OS is defined as the time from initial treatment to death due to any cause.

System evaluation and head and neck cancer examination

Laboratory examination

Before enrollment, the following examination have to be tested: blood routine test, urine routine test, liver and kidney function, glucose test, fasting blood sugar, blood electrolyte, hepatitis B, hepatitis C, syphilis, HIV, electrocardiogram, and heart ultrasound. If HBsAg is positive, HBV-DNA will be checked further.

During and after each treatment cycle: blood routine, liver and kidney function, and other necessary examinations are assessed.

Pathology and biomarker examination

Before enrollment: biopsy histopathology is applied to confirm the squamous cell carcinoma of pharynx, and the expression levels of PD-L1 is evaluated if the biopsy surgery is conducted at Eye & ENT Hospital.

Regarding patients underwent biopsy at Eye & ENT Hospital, routine immunohistochemical diagnosis is performed from biopsy, and further tissue samples are subjected to a series of genetic tests based on the remaining samples, including Whole Exome Sequencing (WES). The analysis content include missense mutations, nonsense mutations, in frame deletion mutations, in frame insertion mutations, frameshift deletions, and frameshift insertions, as well as TMB, P53, MMR, MSI, TLR4/MYD88, MYB/ESPN and NUDT1 that are related to head and neck cancer discovered in our previous studies. These biomarkers are analyzed based on groups including CR/PR, SD/PD, pre-treatment, post-treatment, and TNM staging groups.

At different stages of treatment, blood samples are collected from enrolled patients for whole blood testing and analysis. Using flow cytometry platform to detect the content and proportion of immune cells in each subgroup, we compare them according to grouping conditions, including CR/PR, SD/PD, before and after treatment, as well as TNM staging. Data analysis will be conducted, and the clinical significance will be discussed from the tested significant genes.

The microbiota from swabs samples of this study is also analyzed.

Imaging examination

Before enrollment, the following imaging examination have to be tested: fibrolaryngoscopy of the primary lesion, contrast-enhanced MRI/CT scans of the primary lesion and neck, CT scan of the chest, gastroscopy, ultrasound or CT of abdomen, PET-CT (if possible). MRI/CT scans are conducted at baseline and two months intervals after therapy initiation to evaluate response.

After treatment, the following imaging examination have to be tested: fibrolaryngoscopy once every 1 - 3 months, contrast-enhanced MRI/CT scans of the primary lesion and neck once every six months, ultrasound or CT abdomen once every six months, PET-CT once a year (if possible).

Safety profile evaluation

Laboratory examination and adverse events ((CTCAE) v5.0 are recorded and evaluated as safety profiles by oncologists.

Quality of life score

Karnofsky Performance Status, Zubrod-ECOG-WHO and the European Organization for Research and Treatment of Cancer Quality of Life Questionnaire Head and Neck Module (EORTC-QLQ-H&N30) are applied to evaluate the quality of life of enrolled patients (see appendix).

Peripheral blood and tumor tissue samples

Peripheral blood samples of enrolled patients are obtained during the therapeutic periods of

first, second, and third cycle of induction chemoimmunotherapy, as well as three times of before, during, and after radioimmunotherapy (six blood samples will be obtain during the whole treatment). When a patient undergoing surgical treatment, tissue sample is obtained and preserved in Department of Pathology. When a patient receiving biopsy treatment at Eye & ENT Hospital, the expression of PD-L1 is assessed. The correlation analysis of PD-L1 expression level with ORR and prognosis will be analyzed.

Toxic side effects, treatment discontinue, and dosage adjustment

During radiation period, occurrence and severity of acute radiation toxic side effects should be evaluated weekly, including radiation skin reactions, radiation mucosal reactions, and radiation pneumonia. At the same time, try to avoid interruptions in radiation therapy. If a patient has white membrane fusion or wet peeling skin reactions that is ineffective after relative treatment, severe pharyngodynia leads to more than 15% weight loss and other serious radiation side effects, the treatment of radiotherapy have to be suspended. The reason and time of the discontinue of radiotherapy are documented in detail. Radiotherapy is performed once a day from Monday to Friday. During the treatment period, radiotherapy discontinue less than two times and accumulated time less than 10 days are allowed. During the treatment period, radiation therapy require to be performed as planned rigorously, unless the patient has event of disease progresses or cannot tolerate adverse event caused by radiation therapy, or refuses radiation therapy.

Statistical analysis

The sample size is calculated based on the primary endpoint of ORR, which is predicted to be 80% in this study, and the history control value is 60% from our previous results. With 80% detection power at the formal statistical boundary for the significance of 0.05, 43 participants are required to assess the antitumor activity of camrelizumab, plus 15% of cases due to loss to follow-up. Overall, 51 participants are required to enroll in this trial.

Baseline characteristics, AE, and efficacy follow the intention-to-treat principle in this trial.

The 95% CI and *p* values are provided using the exact binomial distribution. LPR, PFS, MFS, and OS are analyzed by using the intention-to-treat population using the Kaplan-Meier method and Log-rank test. Grade three or worse AEs are evaluated across treatments as a dichotomous variable using patients who receive at least one dose of camrelizumab. A *p*-value less than 0.05 was considered statistically significant. SPSS (version 23.0 IBM SPSS Statistics) or Stata (version 17) is used to analyze all of the data.

Adverse event

An Adverse Event (AE) is any unfavorable and unintended medical occurrence, and the sign (including an abnormal laboratory finding), symptom, or disease temporally associated with the use of a medical treatment or procedure that may or may not be considered related to the medical treatment or (investigational) procedure. The AE is also a term that is a unique representation of a specific event used for medical documentation and scientific analyses.

Grades (Common Terminology Criteria for Adverse Events (CTCAE) v5.0 refers to the severity of the AE. The CTCAE displays grades one to five with unique clinical descriptions of severity for each AE based on the general guideline:

Grade 1 Mild; asymptomatic or mild symptoms; clinical or diagnostic observations only; intervention not indicated.
Grade 2 Moderate; minimal, local or noninvasive intervention indicated; limiting age - appropriate instrumental activities of daily living[*].
Grade 3 Severe or medically significant but not immediately life-threatening; hospitalization or prolongation of hospitalization indicated; disabling; limiting self-care[#] activities of daily living.
Grade 4 Life-threatening consequences; urgent intervention indicated.
Grade 5 Death related to AE.

[*]Daily living activities refer to preparing meals, shopping for groceries or clothes, using the telephone, managing money, etc. [#]Self-care activities of daily living refer to bathing, dressing

and undressing, feeding self, using the toilet, taking medications, and not bedridden.

Serious adverse event

A serious adverse event (SAE) is considered as any undesirable sign, symptom, and medical condition with one or more of the following outcomes: fatal, life-threatening, requiring or prolonging inpatient hospitalization, resulting in persistent or significant disability/incapacity, constituting a congenital malformations or defects, medically significant events which the investigator regards as serious based on appropriate medical judgment, and any serious psychological and emotional distress resulting in study participation (suggesting need for professional counseling or intervention).

A major medical event refers to a situation that does not immediately endanger life, but may pose a threat to patients and may require certain intervention measures to avoid the occurrence of the aforementioned serious consequences.

Adverse event document

During treatment and follow-up, investigators detect AEs through information of inquiry or assessment of examinations. All information about AEs needs to be documented. For SAE, information have to be filled in Serious AE Form. All definitional relevant signs, symptoms, and abnormal diagnostic tests are classified, and a single diagnostic record is made in the case report form (CRF). List the various components of the diagnosis for verification.

All AEs occurring during the observation phase must be recorded and the clinical process of each event followed up until resolved, the situation stabilized, or until it is clear that it was not caused by the study treatment or participation in the trial. Serious AE that still exist at the end of the study period must be followed up to track their final results. Any Serious AE that may be related to the study treatment during the trial period must be recorded and reported in a timely manner.

Follow-up

The first years after treatment, every month

The second year after treatment, every three months

More than two years, every three months

The examination of follow-up include physical examination, imaging examination, and fibrolaryngoscopy of head and neck based on risk of locoregional relapse, second primaries, treatment sequelae, and toxicity. Recording of each follow-up have to be documented.

The relationship between adverse events (AE) and treatment evaluation

The possible relationship between AE and treatment methods of this trial using a five levels classification method (National Medical Products Administration, www.nmpa.gov.cn). The first three levels are judged to be related to the investigational treatment. When calculating the incidence of AEs, the total of the three levels is used as the numerator, and the total number of enrolled subjects is used as the denominator.

Definite: the AE is clearly related to the study treatment.

Probable: the AE is likely related to the study treatment.

Possible: the AE may be related to the study treatment.

Unlikely: the AE is doubtfully related to the study treatment.

Unrelated: the AE is clearly NOT related to the study treatment.

Patient withdrew from the trial

All patients with AEs may withdraw from the trial at any time according to the judgment of the investigators. If a patient withdraws from the trial due to AEs, the CRF and recordings of AEs should be documented.

Ethics

All protocols of this clinical trial study are carried out in accordance with the principles established by the 18th World Medical Assembly (Declaration of Helsinki, 1964) and its subsequent amendments.

The Ethics Committees of Eye & ENT Hospital, Fudan University, approved the trial protocol and treatments.

Patients have to write informed consent before enrollment.

Clinical trial management and quality control

The CRF of every enrolled case has to be documented completely by the investigators. The completed CRF have to be reviewed by the designated investigator and delivered to the main investigators for analysis. If there is any doubt in the verification process, it is necessary to check out with the recording researchers carefully in time. The data have to be input and managed by designated and specialized researchers. After the data input and verification is completed, the data specialized manager, the main researcher and the statistical analyst review it. These investigators complete the final definition and evaluation of the analysis population.

AE monitoring and reporting is a routine part of this clinical trial. In case of SAE, it must be reported and documented in time. If necessary, it can be decided to suspend the study after discussion. The treatments and test examination methods are provided. When serious AEs occur, investigators and relevant departments have to check out carefully in time. They should check the process meets the requirements of GCP and clinical trial plan.

Clinician of this study include the nurse practitioner, registered nurse, pathologist, radiologist, interventional radiologist, surgeon, oncologist, internist, and other medical professionals responsible for the patient’s care. All investigators must be trained before clinical trials carrying out, and all the work under the guidance of senior professionals. The professional medical staff give the subjects medication, understand the medication situation in detail, and ensure the subjects' compliance. The process have to be execute strictly according to clinical

trial plan.

Appendix

Karnofsky Performance Status

Percent	Performance Status
100	Normal no complaints; no evidence of disease
90	Able to carry on normal activity; minor signs or symptoms of disease
80	Normal activity with effort; some signs or symptoms of disease
70	Cares for self; unable to carry on normal activity or to do active work
60	Requires occasional assistance, but is able to care for most of personal needs
50	Requires considerable assistance and frequent medical care
40	Disabled; requires special care and assistance
30	Disabled; requires special care and assistance
20	Very sick; hospital admission necessary; active supportive treatment necessary
10	Very sick; hospital admission necessary; active supportive treatment necessary
0	Deceased

Zubrod-ECOG-WHO

Zubrod-ECOG-WHO runs from 0 to 5, with 0 denoting perfect health and 5 death.

0	Asymptomatic (Fully active, able to carry on all predisease activities without restriction)
1	Symptomatic but completely ambulatory (Restricted in physically strenuous activity but ambulatory and able to carry out work of a light or sedentary nature. For example, light housework, office work)
2	Symptomatic, <50% in bed during the day (Ambulatory and capable of all self care but unable to carry out any work activities. Up and about more than 50% of waking hours)
3	Symptomatic, >50% in bed, but not bedbound (Capable of only limited self-care, confined to bed or chair 50% or more of waking hours)
4	Symptomatic, >50% in bed, but not bedbound (Capable of only limited self-care, confined to bed or chair 50% or more of waking hours)
5	Death

EORTC-QLQ-H&N30

Please fill in your initials:

Your birthdate (Day, Month, Year):

Today's date (Day, Month, Year):

	Not at All	A Little	Quite a Bit	Very Much
1. Do you have any trouble doing strenuous activities, like carrying a heavy shopping bag or a suitcase?	1	2	3	4
2. Do you have any trouble taking a long walk?	1	2	3	4
3. Do you have any trouble taking a short walk outside of the house?	1	2	3	4
4. Do you need to stay in bed or a chair during the day?	1	2	3	4
5. Do you need help with eating, dressing, washing yourself or using the toilet?	1	2	3	4

**During the past week:**	Not at All	A Little	Quite a Bit	Very Much
6. Were you limited in doing either your work or other daily activities?	1	2	3	4
7. Were you limited in pursuing your hobbies or other leisure time activities?	1	2	3	4
8. Were you short of breath?	1	2	3	4
9. Have you had pain?	1	2	3	4
10. Did you need to rest?	1	2	3	4
11. Have you had trouble sleeping?	1	2	3	4
12. Have you felt weak?	1	2	3	4
13. Have you lacked appetite?	1	2	3	4
14. Have you felt nauseated?	1	2	3	4
15. Have you vomited?	1	2	3	4
16. Have you been constipated?	1	2	3	4

**During the past week:**	Not at All	A Little	Quite a Bit	Very Much
17. Have you had diarrhea?	1	2	3	4

18. Were you tired?	1	2	3	4
19. Did pain interfere with your daily activities?	1	2	3	4
20. Have you had difficulty in concentrating on things, like reading a newspaper or watching television?	1	2	3	4
21. Did you feel tense?	1	2	3	4
22. Did you worry?	1	2	3	4
23. Did you feel irritable?	1	2	3	4
24. Did you feel depressed?	1	2	3	4
25. Have you had difficulty remembering things?	1	2	3	4
26. Has your physical condition or medical treatment interfered with your family life?	1	2	3	4
27. Has your physical condition or medical treatment interfered with your social activities?	1	2	3	4
28. Has your physical condition or medical treatment caused you financial difficulties?	1	2	3	4

For the following questions please circle the number between 1 and 7 that best applies to you

29. How would you rate your overall health during the past week?

1 2 3 4 5 6 7

Very poor Excellent

30. How would you rate your overall quality of life during the past week?

1 2 3 4 5 6 7

Very poor Excellent

Final protocol (Version 1.2(2021-08-08))

Background

Head and neck cancer is the sixth most common malignant tumor, and hypopharyngeal cancer is one common and aggressive type of head and neck cancer, displaying inferior prognosis generally[1]. Approximately 80% of patients with hypopharyngeal cancer have locally advanced (stage III and IV) lesions at the time of diagnose, and the 5-year overall survival rate for surgical treatment of locally advanced hypopharyngeal cancer is 30% in China[2]. The current treatments includes a combination of surgery, radiation therapy, chemotherapy, and immunotherapy. Although the surgical approach and radio/chemotherapy regimen continuing reform, the survival rate of patients has not been significantly improved[2]. Additionally, 30% to 40% of patients die due to difficult treatment of recurrence or metastasis despite undergoing radical therapy[1,2]. Therefore, effective clinical treatment plans to preserve laryngeal function and improve survival outcomes have become a challenge for head and neck surgeons and oncologists.

Currently, comprehensive treatment with surgery plus postoperative radio/chemotherapy for advanced head and neck cancer is the main therapy regimen in China. Most advanced laryngeal and hypopharyngeal cancer patients lose their normal pronunciation and respiratory function after total laryngectomy, which brings serious functional impairment and psychological problems to them[3]. In recent years, there have been significant changes in the treatment plans for advanced laryngeal and hypopharyngeal cancer[3]. The important change is derived from the representative clinical trial reported by the Veterans Affairs study, which found that cisplatin+5-fluorouracil induced chemotherapy (PF) plus radiotherapy for patients with advanced laryngeal cancer, without sacrificing the survival rate, to a greater extent preserve the laryngeal function, and improve the treatment of advanced laryngeal cancer to non-surgical treatment[4]. Besides, EORTC 24891 study finds the improved regimen of treatment for hypopharyngeal cancer [5]. Patients receive PF induced chemotherapy, and those who responded effectively receive radical radiotherapy; the rest patients receive surgery and postoperative radiotherapy. There is

no difference in treatment failure rates between these two groups in terms of local, cervical lymph nodes, and secondary primary cancer, but the induction chemotherapy group have a lower rate of distant metastasis and a longer median survival time compared with surgery and postoperative radiotherapy[5]. However, the study by PARADIGM demonstrates that the clinical efficacy of TPF induced chemotherapy combined with radiochemotherapy and concurrent radiochemotherapy in the treatment of advanced head and neck cancer is similar; there is no significant difference in overall survival between these two groups (73% vs 78%)[6]. Induction chemotherapy with TPF, an evidence-based option for advanced HNSCC, establishes a favorable response and milder toxicity profile and improves survival outcomes and function preservation from pooled data analyses (TAX 323/EORTC 24971, TAX 324, GORTEC 2000-01, and TTCC 2002) [7]. Due to different national conditions, these non-surgical treatment plans for larynx preservation have not been widely accepted by many hospitals in the treatment of head and neck cancer in China, and further study is required to confirm the differences among these therapy regimens.

Recently, immune checkpoint inhibitors such as humanized anti-programmed death receptor 1 (PD-1) antibodies have presented efficacy and safety in unresectable head and neck cancer. Patients with recurrent or metastatic disease after platinum chemotherapy treated with nivolumab monoclonal antibody have prolonged overall survival over standard and single-agent therapy treatment.[8] Pembrolizumab monotherapy is well tolerated, and shows clinically substantial antitumor activity in patients with recurrent or metastatic head and neck cancer, with characteristics of prolonging overall survival and favorable safety profile than standard care therapy[9]. Camrelizumab (SHR-1210), another PD-1 monoclonal antibody, is a well-tolerated and appropriate option for patients with recurrent or metastatic nasopharyngeal carcinoma[10]. Camrelizumab monotherapy, gemcitabine, and cisplatin present an acceptable toxicity profile and encourage antitumor activity for patients with treatment-naive recurrent or metastatic nasopharyngeal carcinoma[10]. The discussion of the best practices for head and neck cancer remains ongoing, and the efficacy and safety profile of camrelizumab combined with TPF in head and neck cancer are still unknown.

The purpose of this study is to evaluate the oncological outcome, laryngeal preservation,

and safety profile of camrelizumab in combination with a modified TPF (docetaxel, cisplatin, and capecitabine) regimen as induction therapy for locally advanced hypopharyngeal cancer patients who have previously untreated history and required total laryngectomy.

Reference

1. Chen W, Zheng R, Baade PD, et al. Cancer statistics in China, 2015. CA Cancer J Clin 2016;66(2):115-32.
2. Expert consensus on surgery and comprehensive treatment of hypopharyngeal carcinoma. Zhonghua Er Bi Yan Hou Tou Jing Wai Ke Za Zhi. 2017. 52(1):16-24. doi: 10.3760/cma.j.issn.1673-0860.2017.01.004.
3. Steuer CE, El-Deiry M, Parks JR, Higgins KA, Saba NF. An update on larynx cancer. CA Cancer J Clin 2017;67(1):31-50.
4. Department of Veterans Affairs Laryngeal Cancer Study G, Wolf GT, Fisher SG, et al. Induction chemotherapy plus radiation compared with surgery plus radiation in patients with advanced laryngeal cancer. N Engl J Med 1991;324(24):1685-90.
5. Lefebvre JL, Chevalier D, Luboinski B, Kirkpatrick A, Collette L, Sahmoud T. Larynx preservation in pyriform sinus cancer: preliminary results of a European Organization for Research and Treatment of Cancer phase III trial. EORTC Head and Neck Cancer Cooperative Group. J Natl Cancer Inst 1996;88(13):890-9.
6. Haddad R, O'Neill A, Rabinowits G, et al. Induction chemotherapy followed by concurrent chemoradiotherapy (sequential chemoradiotherapy) versus concurrent chemoradiotherapy alone in locally advanced head and neck cancer (PARADIGM): a randomised phase 3 trial. Lancet Oncol 2013;14(3):257-64.
7. Haddad RI, Posner M, Hitt R, et al. Induction chemotherapy in locally advanced squamous cell carcinoma of the head and neck: role, controversy, and future directions. Ann Oncol 2018;29(5):1130-1140.
8. Ferris RL, Blumenschein G, Jr., Fayette J, et al. Nivolumab for Recurrent Squamous-Cell Carcinoma of the Head and Neck. N Engl J Med 2016;375(19):1856-1867.
9. Seiwert TY, Burtness B, Mehra R, et al. Safety and clinical activity of pembrolizumab for

treatment of recurrent or metastatic squamous cell carcinoma of the head and neck (KEYNOTE-012): an open-label, multicentre, phase 1b trial. Lancet Oncol 2016;17(7):956-965.

10. Fang W, Yang Y, Ma Y, et al. Camrelizumab (SHR-1210) alone or in combination with gemcitabine plus cisplatin for nasopharyngeal carcinoma: results from two single-arm, phase 1 trials. Lancet Oncol 2018;19(10):1338-1350.

Study design

This is an open-label, single-arm, phase II, prospective, multidisciplinary and single-center study, and is sponsored and performed by Department of Otorhinolaryngology Head and Neck surgery and Department Radiation Oncology, Eye & ENT Hospital, Fudan University, Shanghai.

The purpose of this study is to assess antitumor activity and safety profile of camrelizumab (PD-1 inhibitor) plus modified TPF (docetaxel, cisplatin, and capecitabine) of induction therapy for patients with locally advanced hypopharyngeal squamous cell carcinoma (LA HSCC). Enrolled patients involved TNM stage of T3-4aN0-2M0 (AJCC 7th), with types of the pyriform sinus, postcricoid region, and posterior hypopharyngeal wall.

The PD-1 monoclonal antibody is camrelizumab (SHR-1210) from Hengrui Medicine Company, Jiangsu province, China.

Arm and interventions

Single arm with camrelizumab group (induction chemoimmunotherapy)

Induction chemotherapy combined with immunotherapy (TPF + camrelizumab), q3w, three cycles:

TPF chemotherapy

Docetaxel (domestic) 75 mg/m^2 i.v. d1

Cisplatin 25 mg/m^2 i.v. d1-3

Capecitabine 800 mg/m^2 po bid d1-d14

Immunotherapy

Camrelizumab 200 mg i.v. d1

Radical radiotherapy followed by induction chemoimmunotherapy:

Radiotherapy

Intensity-modulated radiation therapy (IMRT). Primary site: GTV dose 66 (2.2 Gy/fraction) - 70 Gy (2 Gy/fraction); CTV 1.6 - 1.9 Gy/fraction. Cervical lymph nodes: radiotherapy plan is the same as the radiotherapy plan of primary lesion.

Concurrent immunotherapy

Camrelizumab 200 mg i.v. d1, d22

Maintenance period

After completing concurrent radiotherapy combined with immunotherapy, camrelizumab 200 mg q3w will be given up to 12 months (calculated from the time of the first dose of PD-1 immunotherapy). The dose of camrelizumab could not be modified.

Surgical treatment

When patients have stable disease (SD) or progress disease (PD) that are evaluated after induction chemoimmunotherapy, they will be assigned to surgical treatment followed by adjuvant (chemo)radiotherapy. If there is further evidence of uncontrolled or progression disease, camrelizumab will be discontinued. Treatment could be discontinued at the patient's or clinician's discretion. Salvage surgery will be conducted if patients have uncontrolled or progressive disease or locoregional recurrence.

Adjuvant therapy

Patients with high-risk factors after total laryngectomy are treated with adjuvant radiotherapy or chemoradiotherapy

Adjuvant chemoradiotherapy

Patients with extranodal extension with or without positive surgical margins receive adjuvant chemoradiotherapy postoperatively.

Adjuvant radiotherapy

Patients with multiple positive nodes (without extranodal extension), perineural invasion, vascular invasion, lymphatic invasion, pT3 or pT4 primary, pN2 or pN3 will receive adjuvant radiotherapy postoperative treatment.

Intensity-modulated radiation therapy (IMRT).

Primary site: GTV dose 66 - 70 Gy: chemoradiotherapy plan, 1.8 - 2 Gy/fraction, radiotherapy plan, 1.8 – 2.2 Gy/fraction.

Cervical lymph nodes: radiotherapy plan is the same as the radiotherapy plan of primary site.

Chemotherapy

Cisplatin 25 mg/m^2 i.v. d1-3, d22-24

Treatment drugs

Docetaxel is a chemotherapy drug

Cisplatin is a chemotherapy drug.

Capecitabine is a chemotherapy drug.

Camrelizumab (SHR-1210) is a humanized anti-PD1 IgG4 monoclonal antibody (Jiangsu Hengrui). Hengrui Medicine Co. partially donated the study drug.

Treatment plan

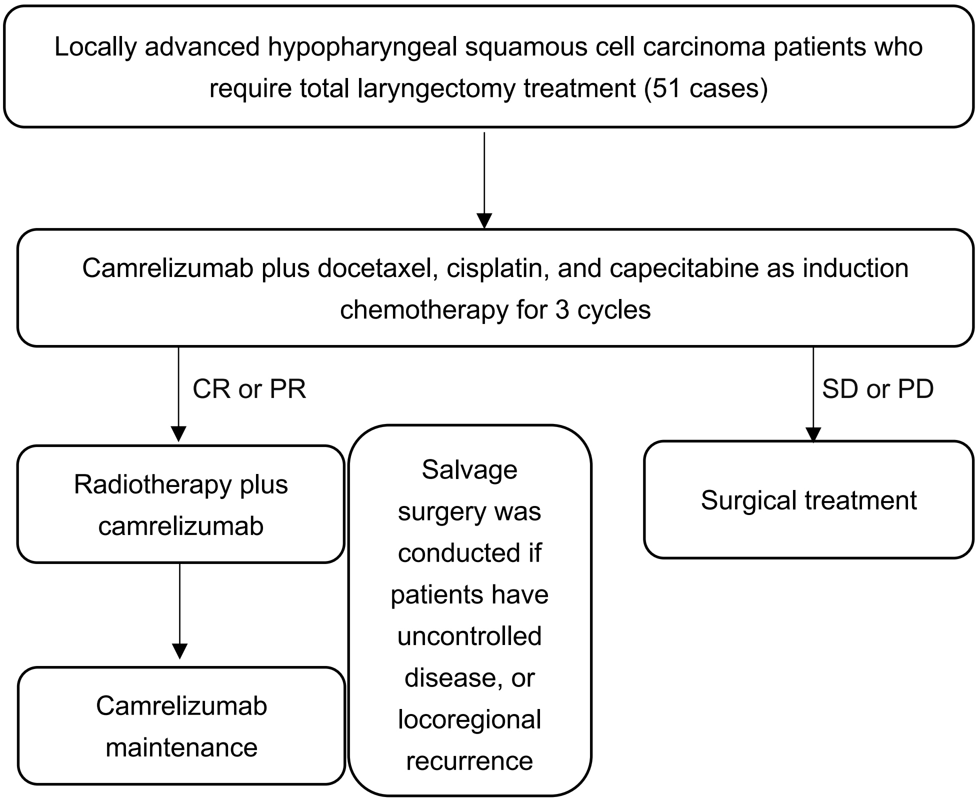

Eligibility criteria

Inclusion criteria:

1. Patients have histologically confirmed hypopharyngeal squamous cell carcinoma and require total laryngectomy, including the piriform fossa, postcricoid region, and posterior pharyngeal wall with TNM stage cT3-4aN0-2M0 (AICC 7th).
2. Able to understand and willing to sign a written informed consent document.
3. Age ≥ 18 and ≤ 70 years.
4. Male or female.
5. Performance status of ECOG 0-2.
6. Expected lifetime > 6 months.
7. Normal blood test, hepatic and renal functions. Normal hearing. Blood test: WBC ≥ 4.0×10^9/L, ANC ≥ 2.0×10^9/L, PLT ≥100×10^9/L, HGB ≥ 100g/ L. Hepatic function: ALT and

AST< upper limit of normal. Kidney function: serum creatinine < upper limit of normal value, and creatinine clearance rate ≥ 60 ml/min Cockcroft-Gault formula. Cardiac ultrasonography left ventricular ejection fraction > 50%.

8. No prior allergic reaction to biological agents and/or ingredient in the drug.
9. No drug abuse.
10. Good compliance.
11. No systemic diseases (such as other tumors, severe heart, lung and central nervous system diseases, etc.).
12. Negative pregnancy test (for female patients with fertility).
13. Male patients with fertility and female patients with fertility and pregnancy risk must agree to use contraceptive methods throughout the study period, and continued until at least 6 months after the last dose of cisplatin and 30 days after the last dose of PD-1 antibody/placebo (whichever occurs later). Female patients do not have fertility (ie meet at least one of the following criteria): underwent hysterectomy and/or bilateral oophorectomy with archival records, medically confirmed ovarian function decline, in postmenopausal state. The Postmenopausal is defined as: at least 12 months of continuous menstruation without other pathological or physiological reasons, and the status confirmed by serum follicle stimulating hormone (FSH) levels is consistent with postmenopausal status.

Exclusion criteria:

1. Patients with cervical lymph node cN3 (AJCC 7th)；
2. Have a history of other cancers in the past five years, patients with radical or untreated prostate cancer (Gleason score ≤ 6), or complete treatment of breast ductal carcinoma in situ, except for cured skin basal cell carcinoma or squamous cell skin cancer.
3. Patients with target lesions who have received radiation therapy or surgery (except biopsy).
4. Patients who have previously used chemotherapy, immunotherapy, or biological targeted therapy for primary tumors.
5. Patients who have participated in other clinical trials within 4 weeks before the test.

6. Any of the following conditions within 6 months before random grouping: myocardial infarction, severe/unstable angina, coronary artery/peripheral artery bypass graft, symptomatic congestive heart failure, cerebrovascular accident, patients with transient ischemic attack or symptomatic pulmonary embolism.
7. Patients with hypertension who cannot control well through single antihypertensive medication (systolic blood pressure >140 mmHg, diastolic blood pressure > 90 mmHg).
8. Patients with grade I or above coronary heart disease, arrhythmia (including men with a QTc interval > 450 ms, women > 470 ms), and cardiac insufficiency.
9. Urinary protein was greater than ++ and 24-hour urinary protein quantification > 1.0 g.
10. Many factors that affect oral medications (such as inability to swallow, nausea, vomiting, chronic diarrhea, and intestinal obstruction).
11. Patients with abnormal coagulation function (INR>1.5, APTT>1.5 ULN), with bleeding tendency.
12. Patients with a history of psychotropic substance abuse that is active or has a mental disorder.
13. Patients who required systemic treatment with corticosteroids (>10 mg prednisone equivalent daily) or other immunosuppressive agents within 2 weeks prior to the first use of the study drug.
14. Patients with a history of severe allergies or allergies; patients with active autoimmune diseases that may worsen when receiving immunostimulants; patients with type 1 diabetes, vitiligo, psoriasis, or hypothyroidism or hyperthyroidism who do not require immunosuppressive therapy are eligible to participate in the study.
15. Patients who have previously been diagnosed with immunodeficiency or known human immunodeficiency virus (HIV) or acquired immunodeficiency syndrome (AIDS)-related diseases. Hepatitis B virus (HBV) or hepatitis C virus (HCV) infection, HBV surface antigen is positive at screening, or patients with positive HCV RNA (ribonucleic acid) when positive for anti-HCV antibody screening test.
16. Vaccination within 4 weeks prior to randomization, except for inactivated vaccines.
17. Pregnant or lactating women who are in the reproductive period but have not taken

effective contraceptive measures.

18. The investigator believes that it is inappropriate for individuals to participate in the trial: having, for example, severe acute or chronic medical conditions (including immune colitis, inflammatory bowel disease, non-infectious pneumonia, pulmonary fibrosis) or mental illness (including recent time (within the past year) or active suicidal ideation or behavior).
19. With a past history of tuberculosis or antituberculosis treatment.

Outcome Measures

Primary outcome measure (time frame: 9 weeks):

Overall response rate (ORR)

ORR is defined as the proportion of patients with best response of complete or partial response (CR or PR) in tumor burden of primary lesion as defined by RECIST 1.1.

The tumor volume system is also used to evaluate the response of three dimensions of the primary lesion during treatment, and PR is defined as volume regression of 70% compared with baseline.

Secondary Outcome Measure (time frame: 3 years):

Larynx preservation rate (LPR)

LPR is defined as the time from initial treatment to a total laryngectomy, dysfunction of the larynx, or death.

Progression free survival (PFS)

PFS is defined as the time from initial treatment to the first documented disease progression or death due to any cause, whichever occurred first.

Metastasis free survival (MFS)

MFS is defined as the time from initial treatment to distant metastasis.

Overall survival (OS)

OS is defined as the time from initial treatment to death due to any cause.

System evaluation and head and neck cancer examination

Laboratory examination

Before enrollment, the following examination have to be tested: blood routine test, urine routine test, liver and kidney function, glucose test, fasting blood sugar, blood electrolyte, hepatitis B, hepatitis C, syphilis, HIV, electrocardiogram, and heart ultrasound. If HBsAg is positive, HBV-DNA will be checked further.

During and after each treatment cycle: blood routine, liver and kidney function, and other necessary examinations are assessed.

Pathological examination

Before enrollment: biopsy histopathology is applied to confirm the squamous cell carcinoma of pharynx, and the expression levels of PD-L1 is evaluated if the biopsy surgery is conducted at Eye & ENT Hospital.

Regarding patients underwent biopsy at Eye & ENT Hospital, routine immunohistochemical diagnosis is performed from biopsy, and further tissue samples are subjected to a series of genetic tests based on the remaining samples, including Whole Exome Sequencing (WES). The analysis content include missense mutations, nonsense mutations, in frame deletion mutations, in frame insertion mutations, frameshift deletions, and frameshift insertions, as well as TMB, P53, MMR, MSI, TLR4/MYD88, MYB/ESPN and NUDT1 that are related to head and neck cancer discovered in our previous studies. These biomarkers are analyzed based on groups including CR/PR, SD/PD, pre-treatment, post-treatment, and TNM staging groups.

At different stages of treatment, blood samples are collected from enrolled patients for whole blood testing and analysis. Using flow cytometry platform to detect the content and proportion of immune cells in each subgroup, we compare them according to grouping conditions, including CR/PR, SD/PD, before and after treatment, as well as TNM staging. Data analysis will be conducted, and the clinical significance will be discussed from the tested significant genes.

The microbiota from swabs samples of this study is also analyzed.

Imaging examination

Before enrollment, the following imaging examination have to be tested: fibrolaryngoscopy of the primary lesion, contrast-enhanced MRI/CT scans of the primary lesion and neck, CT scan of the chest, gastroscopy, ultrasound or CT of abdomen, PET-CT (if possible). MRI/CT scans are conducted at baseline and two months intervals after therapy initiation to evaluate response.

After treatment, the following imaging examination have to be tested: fibrolaryngoscopy once every 1 - 3 months, contrast-enhanced MRI/CT scans of the primary lesion and neck once every six months, ultrasound or CT abdomen once every six months, PET-CT once a year (if possible).

Safety profile evaluation

Laboratory examination and adverse events ((CTCAE) v5.0 are recorded and evaluated as safety profiles by oncologists.

Quality of life score

Karnofsky Performance Status, Zubrod-ECOG-WHO and the European Organization for Research and Treatment of Cancer Quality of Life Questionnaire Head and Neck Module (EORTC-QLQ-H&N30) are applied to evaluate the quality of life of enrolled patients (see appendix).

Peripheral blood and tumor tissue samples

Peripheral blood samples of enrolled patients are obtained during the therapeutic periods of

first, second, and third cycle of induction chemoimmunotherapy, as well as three times of before, during, and after radioimmunotherapy (six blood samples will be obtain during the whole treatment). When a patient undergoing surgical treatment, tissue sample is obtained and preserved in Department of Pathology. When a patient receiving biopsy treatment at Eye & ENT Hospital, the expression of PD-L1 is assessed. The correlation analysis of PD-L1 expression level with ORR and prognosis will be analyzed.

Toxic side effects, treatment discontinue, and dosage adjustment

During radiation period, occurrence and severity of acute radiation toxic side effects should be evaluated weekly, including radiation skin reactions, radiation mucosal reactions, and radiation pneumonia. At the same time, try to avoid interruptions in radiation therapy. If a patient has white membrane fusion or wet peeling skin reactions that is ineffective after relative treatment, severe pharyngodynia leads to more than 15% weight loss and other serious radiation side effects, the treatment of radiotherapy have to be suspended. The reason and time of the discontinue of radiotherapy are documented in detail. Radiotherapy is performed once a day from Monday to Friday. During the treatment period, radiotherapy discontinue less than two times and accumulated time less than 10 days are allowed. During the treatment period, radiation therapy require to be performed as planned rigorously, unless the patient has event of disease progresses or cannot tolerate adverse event caused by radiation therapy, or refuses radiation therapy.

Statistical analysis

The sample size is calculated based on the primary endpoint of ORR, which is predicted to be 80% in this study, and the history control value is 60% from our previous results. With 80% detection power at the formal statistical boundary for the significance of 0.05, 43 participants are required to assess the antitumor activity of camrelizumab, plus 15% of cases due to loss to follow-up. Overall, 51 participants are required to enroll in this trial.

Baseline characteristics, AE, and efficacy follow the intention-to-treat principle in this trial.

The 95% CI and *p* values are provided using the exact binomial distribution. LPR, PFS, MFS, and OS are analyzed by using the intention-to-treat population using the Kaplan-Meier method and Log-rank test. Grade three or worse AEs are evaluated across treatments as a dichotomous variable using patients who receive at least one dose of camrelizumab. A *p*-value less than 0.05 was considered statistically significant. SPSS (version 23.0 IBM SPSS Statistics) or Stata (version 17) is used to analyze all of the data.

Adverse event

An Adverse Event (AE) is any unfavorable and unintended medical occurrence, and the sign (including an abnormal laboratory finding), symptom, or disease temporally associated with the use of a medical treatment or procedure that may or may not be considered related to the medical treatment or (investigational) procedure. The AE is also a term that is a unique representation of a specific event used for medical documentation and scientific analyses.

Grades (Common Terminology Criteria for Adverse Events (CTCAE) v5.0 refers to the severity of the AE. The CTCAE displays grades one to five with unique clinical descriptions of severity for each AE based on the general guideline:

Grade 1 Mild; asymptomatic or mild symptoms; clinical or diagnostic observations only; intervention not indicated.
Grade 2 Moderate; minimal, local or noninvasive intervention indicated; limiting age - appropriate instrumental activities of daily living[*].
Grade 3 Severe or medically significant but not immediately life-threatening; hospitalization or prolongation of hospitalization indicated; disabling; limiting self-care[#] activities of daily living.
Grade 4 Life-threatening consequences; urgent intervention indicated.
Grade 5 Death related to AE.

[*]Daily living activities refer to preparing meals, shopping for groceries or clothes, using the telephone, managing money, etc. [#]Self-care activities of daily living refer to bathing, dressing

and undressing, feeding self, using the toilet, taking medications, and not bedridden.

Serious adverse event

A serious adverse event (SAE) is considered as any undesirable sign, symptom, and medical condition with one or more of the following outcomes: fatal, life-threatening, requiring or prolonging inpatient hospitalization, resulting in persistent or significant disability/incapacity, constituting a congenital malformations or defects, medically significant events which the investigator regards as serious based on appropriate medical judgment, and any serious psychological and emotional distress resulting in study participation (suggesting need for professional counseling or intervention).

A major medical event refers to a situation that does not immediately endanger life, but may pose a threat to patients and may require certain intervention measures to avoid the occurrence of the aforementioned serious consequences.

Adverse event document

During treatment and follow-up, investigators detect AEs through information of inquiry or assessment of examinations. All information about AEs needs to be documented. For SAE, information have to be filled in Serious AE Form. All definitional relevant signs, symptoms, and abnormal diagnostic tests are classified, and a single diagnostic record is made in the case report form (CRF). List the various components of the diagnosis for verification.

All AEs occurring during the observation phase must be recorded and the clinical process of each event followed up until resolved, the situation stabilized, or until it is clear that it was not caused by the study treatment or participation in the trial. Serious AE that still exist at the end of the study period must be followed up to track their final results. Any Serious AE that may be related to the study treatment during the trial period must be recorded and reported in a timely manner.

Follow-up

The first years after treatment, every month

The second year after treatment, every three months

More than two years, every three months

The examination of follow-up include physical examination, imaging examination, and fibrolaryngoscopy of head and neck based on risk of locoregional relapse, second primaries, treatment sequelae, and toxicity. Recording of each follow-up have to be documented.

The relationship between adverse events (AE) and treatment evaluation

The possible relationship between AE and treatment methods of this trial using a five levels classification method (National Medical Products Administration, www.nmpa.gov.cn). The first three levels are judged to be related to the investigational treatment. When calculating the incidence of AEs, the total of the three levels is used as the numerator, and the total number of enrolled subjects is used as the denominator.

Definite: the AE is clearly related to the study treatment.

Probable: the AE is likely related to the study treatment.

Possible: the AE may be related to the study treatment.

Unlikely: the AE is doubtfully related to the study treatment.

Unrelated: the AE is clearly NOT related to the study treatment.

Patient withdrew from the trial

All patients with AEs may withdraw from the trial at any time according to the judgment of the investigators. If a patient withdraws from the trial due to AEs, the CRF and recordings of AEs should be documented.

Ethics

All protocols of this clinical trial study are carried out in accordance with the principles established by the 18th World Medical Assembly (Declaration of Helsinki, 1964) and its subsequent amendments.

The Ethics Committees of Eye & ENT Hospital, Fudan University, approved the trial protocol and treatments.

Patients have to write informed consent before enrollment.

Clinical trial management and quality control

The CRF of every enrolled case has to be documented completely by the investigators. The completed CRF have to be reviewed by the designated investigator and delivered to the main investigators for analysis. If there is any doubt in the verification process, it is necessary to check out with the recording researchers carefully in time. The data have to be input and managed by designated and specialized researchers. After the data input and verification is completed, the data specialized manager, the main researcher and the statistical analyst review it. These investigators complete the final definition and evaluation of the analysis population.

AE monitoring and reporting is a routine part of this clinical trial. In case of SAE, it must be reported and documented in time. If necessary, it can be decided to suspend the study after discussion. The treatments and test examination methods are provided. When serious AEs occur, investigators and relevant departments have to check out carefully in time. They should check the process meets the requirements of GCP and clinical trial plan.

Clinician of this study include the nurse practitioner, registered nurse, pathologist, radiologist, interventional radiologist, surgeon, oncologist, internist, and other medical professionals responsible for the patient's care. All investigators must be trained before clinical trials carrying out, and all the work under the guidance of senior professionals. The professional medical staff give the subjects medication, understand the medication situation in detail, and ensure the subjects' compliance. The process have to be execute strictly according to clinical

trial plan.

Appendix

Karnofsky Performance Status

Percent	Performance Status
100	Normal no complaints; no evidence of disease
90	Able to carry on normal activity; minor signs or symptoms of disease
80	Normal activity with effort; some signs or symptoms of disease
70	Cares for self; unable to carry on normal activity or to do active work
60	Requires occasional assistance, but is able to care for most of personal needs
50	Requires considerable assistance and frequent medical care
40	Disabled; requires special care and assistance
30	Disabled; requires special care and assistance
20	Very sick; hospital admission necessary; active supportive treatment necessary
10	Very sick; hospital admission necessary; active supportive treatment necessary
0	Deceased

Zubrod-ECOG-WHO

Zubrod-ECOG-WHO runs from 0 to 5, with 0 denoting perfect health and 5 death.

0	Asymptomatic (Fully active, able to carry on all predisease activities without restriction)
1	Symptomatic but completely ambulatory (Restricted in physically strenuous activity but ambulatory and able to carry out work of a light or sedentary nature. For example, light housework, office work)
2	Symptomatic, <50% in bed during the day (Ambulatory and capable of all self care but unable to carry out any work activities. Up and about more than 50% of waking hours)
3	Symptomatic, >50% in bed, but not bedbound (Capable of only limited self-care, confined to bed or chair 50% or more of waking hours)
4	Symptomatic, >50% in bed, but not bedbound (Capable of only limited self-care, confined to bed or chair 50% or more of waking hours)
5	Death

EORTC-QLQ-H&N30

Please fill in your initials:
Your birthdate (Day, Month, Year):
Today's date (Day, Month, Year):

	Not at All	A Little	Quite a Bit	Very Much
1. Do you have any trouble doing strenuous activities, like carrying a heavy shopping bag or a suitcase?	1	2	3	4
2. Do you have any trouble taking a long walk?	1	2	3	4
3. Do you have any trouble taking a short walk outside of the house?	1	2	3	4
4. Do you need to stay in bed or a chair during the day?	1	2	3	4
5. Do you need help with eating, dressing, washing yourself or using the toilet?	1	2	3	4

**During the past week:**	Not at All	A Little	Quite a Bit	Very Much
6. Were you limited in doing either your work or other daily activities?	1	2	3	4
7. Were you limited in pursuing your hobbies or other leisure time activities?	1	2	3	4
8. Were you short of breath?	1	2	3	4
9. Have you had pain?	1	2	3	4
10. Did you need to rest?	1	2	3	4
11. Have you had trouble sleeping?	1	2	3	4
12. Have you felt weak?	1	2	3	4
13. Have you lacked appetite?	1	2	3	4
14. Have you felt nauseated?	1	2	3	4
15. Have you vomited?	1	2	3	4
16. Have you been constipated?	1	2	3	4

**During the past week:**	Not at All	A Little	Quite a Bit	Very Much
17. Have you had diarrhea?	1	2	3	4

18. Were you tired?	1	2	3	4
19. Did pain interfere with your daily activities?	1	2	3	4
20. Have you had difficulty in concentrating on things, like reading a newspaper or watching television?	1	2	3	4
21. Did you feel tense?	1	2	3	4
22. Did you worry?	1	2	3	4
23. Did you feel irritable?	1	2	3	4
24. Did you feel depressed?	1	2	3	4
25. Have you had difficulty remembering things?	1	2	3	4
26. Has your physical condition or medical treatment interfered with your family life?	1	2	3	4
27. Has your physical condition or medical treatment interfered with your social activities?	1	2	3	4
28. Has your physical condition or medical treatment caused you financial difficulties?	1	2	3	4

For the following questions please circle the number between 1 and 7 that best applies to you

31. How would you rate your overall health during the past week?

1 2 3 4 5 6 7

Very poor Excellent

32. How would you rate your overall quality of life during the past week?

1 2 3 4 5 6 7

Very poor Excellent

Summary of changes – Protocol

Protocol version: 1.1(2019-07-26)
Protocol date: 2019-07-26

For protocol amendment # to:
Protocol version: 1.2(2021-08-08)
Protocol date: 2021-08-08

#	Section	Changes	
		Previous version<br>Version 1.1(2019-07-26)<br>Date (2019-07-26)	Modified version<br>Version 1.2(2021-08-08)<br>Date (2021-08-08)
1	Inclusion criteria	5) No distant metastasis, M0.	Delete this criteria, due to this criteria mentioned in “criteria 1” (M0)
2	Inclusion criteria	9) No prior allergic reaction to apatinib/biological agents and/or ingredient in the drug.	8) No prior allergic reaction to biological agents and/or ingredient in the drug (due to the criteria 5 mentioned in criteria 1 (M0), and it was deleted. The “criteria 9)” was became “criteria 8)”).
3	Exclusion criteria	/	19) With a past history of tuberculosis or antituberculosis treatment (new one added).
4	Inclusion criteria	cT3-4aN1-2M0 (AJCC 7th)	cT3-4aN0-2M0 (AJCC 7th)
5	Arms and interventions	Maintenance period<br>After completing concurrent chemoradiotherapy combined with immunotherapy, camrelizumab 200 mg q3w and apatinib 250 mg qd will be given every three weeks up to 18 cycles (calculated from the time of the first dose of PD-1 immunotherapy).	Maintenance period<br>After completing concurrent chemoradiotherapy combined with immunotherapy, camrelizumab 200 mg q3w will be given every three weeks up to 18 cycles (calculated from the time of the first dose of PD-1 immunotherapy).

Original statistical analysis plan (version 1.1(2019-07-26))

Study design

This is an open-label, single-arm, phase II, prospective, multidisciplinary and single-center study, and is sponsored and performed by Department of Otorhinolaryngology Head and Neck surgery and Department Radiation Oncology, Eye & ENT Hospital, Fudan University, Shanghai.

The purpose of this study is to assess antitumor activity and safety profile of camrelizumab (PD-1 inhibitor) and plus TPF of induction therapy for patients with locally advanced hypopharyngeal squamous cell carcinoma (LA HSCC). Enrolled patients involved TNM stage of cT3-4aN1-2M0 (AJCC 7th), with types of the pyriform sinus, postcricoid region, and posterior hypopharyngeal wall.

Primary outcome measure

Overall response rate (ORR)
ORR is defined as the proportion of patients with best response of complete or partial response (CR or PR) in tumor burden of primary lesion as defined by RECIST 1.1. The tumor volume system is also used to evaluate the response of three dimensions of the primary lesion during treatment, and PR is defined as volume regression of 70% compared with baseline.

Secondary outcome measures (time frame: 3 years)

Larynx preservation rate (LPR)
LPR is defined as the time from initial treatment to a total laryngectomy, dysfunction of the larynx, or death.
Progression free survival (PFS)
PFS is defined as the time from initial treatment to the first documented disease progression or death due to any cause, whichever occurred first.
Metastasis free survival (MFS)
MFS is defined as the time from initial treatment to distant metastasis.
Overall survival (OS)
OS is defined as the time from initial treatment to death due to any cause.

Baseline clinical characteristics

Baseline clinical characteristics of 51 patients with LA HSCC are analyzed

Age (years)
  Mean
  Range

Sex
- Male
- Female

Smoking
- Smoking
- No smoking

Drinking
- Drinking
- No Drinking

Tumor site
- Pyriform sinus
- Postcricoid
- Posterior hypopharyngeal wall

T stages 7th
- T3
- T4

N stages
- N0
- N1
- N2b
- N2c
- N3

Clinical stages 7th
- III
- IVa
- IVb

T stages 8th
- T3
- T4

N stages 8 th
- N0
- N1
- N2b
- N2c
- N3

Clinical stages 8th
- III
- IVa
- IVb

ENE
- ENE +
- ENE -

Esophagus involved
- Involved

No involved
Thyroid cartilage
Involved
No involved
Vocal cord mobility
Mobility
Impaired
Fixation
CPS
< 1%
> 1%

Adverse events

Adverse events (grades 1 - 4) in the intention-to-treat population are analyzed
Induced chemoimmunotherapy related adverse events

The whole treatment (induced chemoimmunotherapy + radioimmunotherapy + immunotherapy maintenance) related adverse events

Oncologic outcomes

Laryngeal preservation rate (LPR), progression-free survival (PFS), metastasis-free survival (MFS) and overall survival (OS) in subgroups are analyzed
3-year LPR, 3-year PFS, 3-year MFS, and 3-year OS.
The difference of LPR rate among N stage 8th and 7th.
The difference of LPR rate in T3 stage and T4 stage 8th and 7th.
The difference of LPR rate among N stages 8th and 7th.
The difference of LPR rate among III, IVa and IVb stages.
The difference of LPR among the tumor sites of pyriform sinus, postcricoid, and posterior hypopharyngeal wall.
The difference of PFS rate among N stage 8th and 7th.
The difference of PFS rate in T3 stage and T4 stage8th and 7th.
The difference of PFS rate among III, IVa and IVb stages8th and 7th.
The difference of PFS rate among N stages 8th and 7th.
The difference of PFS among the tumor sites of pyriform sinus, postcricoid, and posterior hypopharyngeal wall.
The difference of MFS rate among N stage 8th and 7th.
The difference of MFS rate in T3 stage and T4 stage 8th and 7th.
The difference of MFS rate among III, IVa and IVb stages8th and 7th.
The difference of MFS rate among N stages 8th and 7th.
The difference of MFS among the tumor sites of pyriform sinus, postcricoid, and posterior

hypopharyngeal wall.

The difference of OS rate among N stage 8th and 7th.

The difference of OS rate in T3 stage and T4 stage 8th and 7th.

The difference of OS rate among III, IVa and IVb stages 8th and 7th.

The difference of OS rate among N stages 8th and 7th.

The difference of OS among the tumor sites of pyriform sinus, postcricoid, and posterior hypopharyngeal wall.

PD-L1 expression in subgroups are analyzed

The ORR levels in group of patients with CPS < 1% and group of patients with CPS ≥ 1%

The expression levels of PD-L1 in the primary tumor site of patients with CR, PR and SD.

Statistical analyses overview

The data cutoff analyses for the present analysis is the day when all patients are enrolled. Baseline characteristics, adverse events and efficacy followed the intention-to-treat principle in this trials. The proportion of patients with ORR is evaluated by investigator per tumor diameter (RECIST (version 1.1) and volume system, and *p* values are provided using exact binomial distribution. LPR, PFS, MFS, and OS are analyzed based on using the intention-to-treat population using Kaplan-Meier method and Log rank test. Grade three or worse adverse events are evaluated across treatments as a dichotomous variable using patients who receive at least one dose of camrelizumab. A *p*-value less than 0.05 is considered statistically significant. Stata or SPSS are applied to analyze all of the data, and the commands of Stata were displayed.

Besides, post-hoc analysis about oncologic outcomes, tissue samples and blood samples of enrolled patients will be perform.

Sample size calculation

The primary endpoint of ORR, which is predicted to be 80% in this study, and the history control value of ORR is 60% in our clinical center. With 80% detection power at the formal statistical boundary for the significance of 0.05, 43 participants are required to assess the antitumor activity of camrelizumab, plus 15% of cases due to loss to follow-up. Overall, 51 cases are required to enroll in this trial.

Baseline clinical characteristics analyses

Describe the baseline clinical characteristics of 51 patients with LA HSCC (Stata commands presented)

summarize Age, detail

tabulate Sex
proportion Sex

tabulate Smoking
proportion Smoking

tabulate Drinking
proportion Drinking

tabulate Drinking
proportion Drinking

tabulate Tumor_sites
proportion Tumor_sites

tabulate T_7th
proportion T_7th

tabulate N_7th
proportion N_7th

tabulate Clinical_stages_7th
proportion Clinical_stages_7th

tabulate T_8th
proportion T_8th

tabulate N_8th

proportion N_8th

tabulate Clinical_stages_8th
proportion Clinical_stages_8th

tabulate ENE
proportion ENE

tabulate Esophagus_involved
proportion Esophagus_involved

Adverse events evaluation

Describe induced chemoimmunotherapy related adverse events in the intention-to-treat population (Stata commands presented)
tabulate adverse events,1,2,3
proportion adverse events,1,2,3

Describe the whole treatment (induced chemoimmunotherapy + radioimmunotherapy + immunotherapy maintenance) related adverse events (Stata commands presented)
tabulate adverse events,1,2,3
proportion adverse events,1,2,3

Figures

Figure: tumor response after induced chemoimmunotherapy in 51 LA HSCC patients from tumor diameter evaluation (Stata commands presented).

twoway (bar diameterevaluation ID), yline(-70) ylabel(-100(20)0) xlabel(#51) xscale(alt)

Figure: tumor response after induced chemoimmunotherapy in 51 LA HSCC patients from tumor volume evaluation (Stata commands presented).

twoway (bar volumeevaluation ID), yline(-70) ylabel(-100(20)0) xlabel(#51) xscale(alt)

Figure: Kaplan-Meier estimates of laryngeal preservation rate of 51 patients with LA HSCC (Stata commands presented).

stset LPR_month, failure(LPR_event==1)
sts graph, risktable censored(single) plotopts(recast(line)) xlabel(0(12)36)

Figure: Kaplan-Meier estimates of laryngeal preservation rate of T3 stage and T4 stage patients with LA HSCC 8th (Stata commands presented).

stset LPR_month, failure(LPR_event==1) scale(1)
sts graph, by(T_8th) risktable censored(single)
sts test T_8th

Figure: Kaplan-Meier estimates of laryngeal preservation rate of T3 stage and T4 stage patients with LA HSCC 7th (Stata commands presented).

stset LPR_month, failure(LPR_event==1) scale(1)
sts graph, by(T_7th) risktable censored(single)
sts test T_7th

Figure: Kaplan-Meier estimates of progression-free survival of 51 patients with LA HSCC (Stata commands presented).

stset PFS_month, failure(PFS_event==1)
sts graph, risktable censored(single) plotopts(recast(line)) xlabel(0(12)36)

Figure: Kaplan-Meier estimates of progression-free survival of T3 stage and T4 stage patients with LA HSCC 8th (Stata commands presented).

stset PFS_month, failure(PFS_event==1) scale(1)
sts graph, by(T_8th) risktable censored(single)
sts test T_8th

Figure: Kaplan-Meier estimates of progression-free survival of T3 stage and T4 stage patients with LA HSCC 7th (Stata commands presented).

stset PFS_month, failure(PFS_event==1) scale(1)
sts graph, by(T_7th) risktable censored(single)
sts test T_7th

Figure: Kaplan-Meier estimates of metastasis-free survival of 51 patients with LA HSCC (Stata commands presented).

stset MFS_month, failure(MFS_event==1)

sts graph, risktable censored(single) plotopts(recast(line)) xlabel(0(12)36),48,60,72

Figure: Kaplan-Meier estimates of metastasis-free survival of T3 stage and T4 stage patients with LA HSCC 8th (Stata commands presented).

stset MFS_month, failure(MFS_event==1) scale(1)

sts graph, by(T_8th) risktable censored(single)

sts test T_8th

Figure: Kaplan-Meier estimates of metastasis-free survival of T3 stage and T4 stage patients with LA HSCC 7th (Stata commands presented).

stset MFS_month, failure(MFS_event==1) scale(1)

sts graph, by(T_7th) risktable censored(single)

sts test T_7th

Figure: Kaplan-Meier estimates of overall survival of 51 patients with LA HSCC (Stata commands presented).

stset OS_month, failure(OS_event==1)

sts graph, risktable censored(single) plotopts(recast(line)) xlabel(0(12)36),48,60,72

Figure: Kaplan-Meier estimates of overall survival of T3 stage and T4 stage patients with LA HSCC 8th (Stata commands presented).

stset OS_month, failure(OS_event==1) scale(1)

sts graph, by(T_8th) risktable censored(single)

sts test T_8th

Figure: Kaplan-Meier estimates of overall survival of T3 stage and T4 stage patients with LA HSCC 7th (Stata commands presented).

stset OS_month, failure(OS_event==1) scale(1)

sts graph, by(T_7th) risktable censored(single)

sts test T_7th

Figure: treatment procedures and time frame of chemoimmunotherapy, radioimmunotherapy, and immunotherapy maintenance of 51 patients (Stata commands presented).

twoway (scatter ID Events)

Subgroups analyses

The difference of laryngeal preservation rate among III, IVa and IVb stages 8th (Stata commands presented).

stset LPR_month, failure(LPR_event==1) scale(1)
sts graph, by(Clinical_stages_8th) risktable censored(single)
sts test Clinical_stages_8th

The difference of laryngeal preservation rate among III, IVa and IVb stages 7th (Stata commands presented).

stset LPR_month, failure(LPR_event==1) scale(1)
sts graph, by(Clinical_stages_7th) risktable censored(single)
sts test Clinical_stages_7th

The difference of laryngeal preservation rate among N stages 8th (Stata commands presented).

stset LPR_month, failure(LPR_event==1) scale(1)
sts graph, by(N_8th) risktable censored(single)
sts test N_8th

The difference of laryngeal preservation rate among N stages 7th (Stata commands presented).

stset LPR_month, failure(LPR_event==1) scale(1)
sts graph, by(N_7th) risktable censored(single)
sts test N_7th

The difference in laryngeal preservation rate between T3 stage and T4 stage patients 8th (Stata commands presented).

stset LPR_month, failure(LPR_event==1) scale(1)
sts graph, by(T_8th) risktable censored(single)
sts test T_8th

The difference in laryngeal preservation rate between T3 stage and T4 stage patients 7th (Stata commands presented).

stset LPR_month, failure(LPR_event==1) scale(1)
sts graph, by(T_7th) risktable censored(single)
sts test T_7th

The difference of laryngeal preservation rate among the tumor sites of the pyriform sinus, postcricoid, and posterior hypopharyngeal wall (Stata commands presented).

stset LPR_month, failure(LPR_event==1) scale(1)
sts graph, by(Tumor_sites) risktable censored(single)
sts test Tumor_sites

The difference of progression-free survival rate among N stages 8th (Stata commands presented).

stset PFS_month, failure(PFS_event==1) scale(1)
sts graph, by(N_8th) risktable censored(single)
sts test N_8th

The difference of progression-free survival rate among N stages 7th (Stata commands presented).

stset PFS_month, failure(PFS_event==1) scale(1)
sts graph, by(N_7th) risktable censored(single)
sts test N_7th

The difference in progression-free survival rate between T3 stage and T4 stage patients 8th (Stata commands presented).

stset PFS_month, failure(PFS_event==1) scale(1)
sts graph, by(T_8th) risktable censored(single)
sts test T_8th

The difference in progression-free survival rate between T3 stage and T4 stage patients 7th (Stata commands presented).

stset PFS_month, failure(PFS_event==1) scale(1)
sts graph, by(T_7th) risktable censored(single)
sts test T_7th

The difference in progression-free survival rate was detected among III, IVa and IVb stages 8th (Stata commands presented).

stset PFS_month, failure(PFS_event==1) scale(1)
sts graph, by(Clinical_stages_8th) risktable censored(single)
sts test Clinical_stages_8th

The difference in progression-free survival rate was detected among III, IVa and IVb stages 7th (Stata commands presented).

stset PFS_month, failure(PFS_event==1) scale(1)
sts graph, by(Clinical_stages_7th) risktable censored(single)
sts test Clinical_stages_7th

The difference of progression-free survival rate among tumor sites of the pyriform sinus, postcricoid, and posterior hypopharyngeal wall (Stata commands presented).

stset PFS_month, failure(PFS_event==1) scale(1)
sts graph, by(Tumor_sites) risktable censored(single)
sts test Tumor_sites

The difference of metastasis-free survival rate among N stages 8th (Stata commands

presented).

stset MFS_month, failure(MFS_event==1) scale(1)
sts graph, by(N_8th) risktable censored(single)
sts test N_8th

The difference of metastasis-free survival rate among N stages 7th (Stata commands presented).

stset MFS_month, failure(MFS_event==1) scale(1)
sts graph, by(N_7th) risktable censored(single)
sts test N_7th

The difference of metastasis-free survival rate between T3 stage and T4 stage patients 8th (Stata commands presented).

stset MFS_month, failure(MFS_event==1) scale(1)
sts graph, by(T_8th) risktable censored(single)
sts test T_8th

The difference of metastasis-free survival rate between T3 stage and T4 stage patients 7th (Stata commands presented).

stset MFS_month, failure(MFS_event==1) scale(1)
sts graph, by(T_7th) risktable censored(single)
sts test T_7th

The difference of metastasis-free survival rate among III, IVa and IVb stages 8th (Stata commands presented).

stset MFS_month, failure(MFS_event==1) scale(1)
sts graph, by(Clinical_stages_8th) risktable censored(single)
sts test Clinical_stages_8th

The difference of metastasis-free survival rate among III, IVa and IVb stages 7th (Stata commands presented).

stset MFS_month, failure(MFS_event==1) scale(1)
sts graph, by(Clinical_stages_7th) risktable censored(single)
sts test Clinical_stages_7th

The difference of metastasis-free survival rate among tumor sites of the pyriform sinus, postcricoid, and posterior hypopharyngeal wall (Stata commands presented).

stset MFS_month, failure(MFS_event==1) scale(1)
sts graph, by(Tumor_sites) risktable censored(single)
sts test Tumor_sites

The difference of overall survival rate among N stages 8th (Stata commands presented).

stset OS_month, failure(OS_event==1) scale(1)
sts graph, by(N_8th) risktable censored(single)

sts test N_8th

The difference of overall survival rate among N stages 7th (Stata commands presented).
stset OS_month, failure(OS_event==1) scale(1)
sts graph, by(N_7th) risktable censored(single)
sts test N_7th

The difference of overall survival rate between T3 stage and T4 stage patients 8th (Stata commands presented).
stset OS_month, failure(OS_event==1) scale(1)
sts graph, by(T_8th) risktable censored(single)
sts test T_8th

The difference of overall survival rate between T3 stage and T4 stage patients 7th (Stata commands presented).
stset OS_month, failure(OS_event==1) scale(1)
sts graph, by(T_7th) risktable censored(single)
sts test T_7th

The difference of overall survival rate among III, IVa and IVb stages 8th (Stata commands presented).
stset OS_month, failure(OS_event==1) scale(1)
sts graph, by(Clinical_stages_8th) risktable censored(single)
sts test Clinical_stages_8th

The difference of overall survival rate among III, IVa and IVb stages 7th (Stata commands presented).
stset OS_month, failure(OS_event==1) scale(1)
sts graph, by(Clinical_stages_7th) risktable censored(single)
sts test Clinical_stages_7th

The difference of overall survival rate among tumor sites of the pyriform sinus, postcricoid, and posterior hypopharyngeal wall (Stata commands presented).
stset OS_month, failure(OS_event==1) scale(1)
sts graph, by(Tumor_sites) risktable censored(single)
sts test Tumor_sites

Estimated 1-year, 2-year and 3-year LPR rates (Stata commands presented).
ltable LPR_month LPR_event, survival failure intervals(12)

Estimated 1-year, 2-year and 3-year PFS rates (Stata commands presented).
ltable PFS_month PFS_event, survival failure intervals(12)

Estimated 1-year, 2-year and 3-year MFS rates (Stata commands presented).

ltable MFS_month MFS_event, survival failure intervals(12)

Rstimated 1-year, 2-year and 3-year OS rates (Stata commands presented).
ltable OS_month OS_event, survival failure intervals(12)

The ORR levels of tumor volume in group of patients with CPS < 1% CPS ≥ 1% (Stata commands presented).
tabstat VolumeResponse_detail, statistics(mean sd) by(PDL1_express) columns(variables)
anova VolumeResponse_detail PDL1_express

The ORR levels of tumor diameter in group of patients with CPS < 1% CPS ≥ 1% (Stata commands presented).
tabstat DiameterResponse_detail, statistics(mean sd) by(PDL1_express) columns(variables)
anova DiameterResponse_detail PDL1_express

The expression results of PD-L1 in the primary tumor site of patients with PR and SD patients (Stata commands presented).
tabulate Volume_evaluation PDL1_express, chi2 column row

Besides, post-hoc analysis about oncologic outcomes, tissue samples and blood samples of enrolled patients will be perform. The statistical analysis will be performed by Stata software and other methods.

Final statistical analysis plan (Version 1.2(2021-08-08))

Study design

This is an open-label, single-arm, phase II, prospective, multidisciplinary and single-center study, and is sponsored and performed by Department of Otorhinolaryngology Head and Neck surgery and Department Radiation Oncology, Eye & ENT Hospital, Fudan University, Shanghai.

The purpose of this study is to assess antitumor activity and safety profile of camrelizumab (PD-1 inhibitor) and plus TPF of induction therapy for patients with locally advanced hypopharyngeal squamous cell carcinoma (LA HSCC). Enrolled patients involved TNM stage of cT3-4aN0-2M0 (AJCC 7th), with types of the pyriform sinus, postcricoid region, and posterior hypopharyngeal wall.

Primary outcome measure (time frame: 9 weeks)

Overall response rate (ORR)
ORR is defined as the proportion of patients with best response of complete or partial response (CR or PR) in tumor burden of primary lesion as defined by RECIST 1.1. The tumor volume system is also used to evaluate the response of three dimensions of the primary lesion during treatment, and PR is defined as volume regression of 70% compared with baseline.

Secondary outcome measures (time frame: 3 years)

Larynx preservation rate (LPR)
LPR is defined as the time from initial treatment to a total laryngectomy, dysfunction of the larynx, or death.
Progression free survival (PFS)
PFS is defined as the time from initial treatment to the first documented disease progression or death due to any cause, whichever occurred first.
Metastasis free survival (MFS)
MFS is defined as the time from initial treatment to distant metastasis.
Overall survival (OS)
OS is defined as the time from initial treatment to death due to any cause.

Baseline clinical characteristics

Baseline clinical characteristics of 51 patients with LA HSCC are analyzed
Age (years)
  Mean
  Range

Sex
- Male
- Female

Smoking
- Smoking
- No smoking

Drinking
- Drinking
- No Drinking

Tumor site
- Pyriform sinus
- Postcricoid
- Posterior hypopharyngeal wall

T stages 7th
- T3
- T4

N stages
- N0
- N1
- N2b
- N2c
- N3

Clinical stages 7th
- III
- IVa
- IVb

T stages 8th
- T3
- T4

N stages 8 th
- N0
- N1
- N2b
- N2c
- N3

Clinical stages 8th
- III
- IVa
- IVb

ENE
- ENE +
- ENE -

Esophagus involved
- Involved

No involved
Thyroid cartilage
Involved
No involved
Vocal cord mobility
Mobility
Impaired
Fixation
CPS
< 1%
> 1%

Adverse events

Adverse events (grades 1 - 4) in the intention-to-treat population are analyzed
Induced chemoimmunotherapy related adverse events

The whole treatment (induced chemoimmunotherapy + radioimmunotherapy + immunotherapy maintenance) related adverse events

Oncologic outcomes

Laryngeal preservation rate (LPR), progression-free survival (PFS), metastasis-free survival (MFS) and overall survival (OS) in subgroups are analyzed
3-year LPR, 3-year PFS, 3-year MFS, and 3-year OS.
The difference of LPR rate among N stage 8th and 7th.
The difference of LPR rate in T3 stage and T4 stage 8th and 7th.
The difference of LPR rate among N stages 8th and 7th.
The difference of LPR rate among III, IVa and IVb stages.
The difference of LPR among the tumor sites of pyriform sinus, postcricoid, and posterior hypopharyngeal wall.
The difference of PFS rate among N stage 8th and 7th.
The difference of PFS rate in T3 stage and T4 stage8th and 7th.
The difference of PFS rate among III, IVa and IVb stages8th and 7th.
The difference of PFS rate among N stages 8th and 7th.
The difference of PFS among the tumor sites of pyriform sinus, postcricoid, and posterior hypopharyngeal wall.
The difference of MFS rate among N stage 8th and 7th.
The difference of MFS rate in T3 stage and T4 stage 8th and 7th.
The difference of MFS rate among III, IVa and IVb stages8th and 7th.
The difference of MFS rate among N stages 8th and 7th.
The difference of MFS among the tumor sites of pyriform sinus, postcricoid, and posterior

hypopharyngeal wall.
The difference of OS rate among N stage 8th and 7th.
The difference of OS rate in T3 stage and T4 stage 8th and 7th.
The difference of OS rate among III, IVa and IVb stages 8th and 7th.
The difference of OS rate among N stages 8th and 7th.
The difference of OS among the tumor sites of pyriform sinus, postcricoid, and posterior hypopharyngeal wall.

PD-L1 expression in subgroups are analyzed
The ORR levels in group of patients with CPS < 1% and group of patients with CPS ≥ 1%
The expression levels of PD-L1 in the primary tumor site of patients with CR, PR and SD.

Statistical analyses overview

The data cutoff analyses for the present analysis is the day when all patients are enrolled. Baseline characteristics, adverse events and efficacy followed the intention-to-treat principle in this trials. The proportion of patients with ORR is evaluated by investigator per tumor diameter (RECIST (version 1.1) and volume system, and *p* values are provided using exact binomial distribution. LPR, PFS, MFS, and OS are analyzed based on using the intention-to-treat population using Kaplan-Meier method and Log rank test. Grade three or worse adverse events are evaluated across treatments as a dichotomous variable using patients who receive at least one dose of camrelizumab. A *p*-value less than 0.05 is considered statistically significant. Stata (version 17) or SPSS (version 23.0 IBM SPSS Statistics) are applied to analyze all of the data, and the commands of Stata were displayed.

Besides, post-hoc analysis about oncologic outcomes, tissue samples and blood samples will be perform.

Sample size calculation

The primary endpoint of ORR, which is predicted to be 80% in this study, and the history control value of ORR is 60% in our clinical center. With 80% detection power at the formal statistical boundary for the significance of 0.05, 43 participants are required to assess the antitumor activity of camrelizumab, plus 15% of cases due to loss to follow-up. Overall, 51 cases are required to enroll in this trial.

power oneproportion 0.6 0.8

Baseline clinical characteristics analyses

Describe the baseline clinical characteristics of 51 patients with LA HSCC (Stata commands presented)
summarize Age, detail

tabulate Sex
proportion Sex

tabulate Smoking
proportion Smoking

tabulate Drinking
proportion Drinking

tabulate Drinking
proportion Drinking

tabulate Tumor_sites
proportion Tumor_sites

tabulate T_7th
proportion T_7th

tabulate N_7th
proportion N_7th

tabulate Clinical_stages_7th
proportion Clinical_stages_7th

tabulate T_8th
proportion T_8th

tabulate N_8th
proportion N_8th

tabulate Clinical_stages_8th
proportion Clinical_stages_8th

tabulate ENE
proportion ENE

tabulate Esophagus_involved
proportion Esophagus_involved

Adverse events evaluation

Describe induced chemoimmunotherapy related adverse events in the intention-to-treat population (Stata commands presented)
tabulate adverse events,1,2,3
proportion adverse events,1,2,3

Describe the whole treatment (induced chemoimmunotherapy + radioimmunotherapy + immunotherapy maintenance) related adverse events (Stata commands presented)
tabulate adverse events,1,2,3
proportion adverse events,1,2,3

Figures

Figure: tumor response after induced chemoimmunotherapy in 51 LA HSCC patients from tumor diameter evaluation (Stata commands presented).

twoway (bar diameterevaluation ID), yline(-70) ylabel(-100(20)0) xlabel(#51) xscale(alt)

Figure: tumor response after induced chemoimmunotherapy in 51 LA HSCC patients from tumor volume evaluation (Stata commands presented).

twoway (bar volumeevaluation ID), yline(-70) ylabel(-100(20)0) xlabel(#51) xscale(alt)

Figure: Kaplan-Meier estimates of laryngeal preservation rate of 51 patients with LA HSCC (Stata commands presented).

stset LPR_month, failure(LPR_event==1)
sts graph, risktable censored(single) plotopts(recast(line)) xlabel(0(12)36),48,60,72

Figure: Kaplan-Meier estimates of laryngeal preservation rate of T3 stage and T4 stage patients with LA HSCC 8th (Stata commands presented).

stset LPR_month, failure(LPR_event==1) scale(1)
sts graph, by(T_8th) risktable censored(single)
sts test T_8th

Figure: Kaplan-Meier estimates of laryngeal preservation rate of T3 stage and T4 stage patients with LA HSCC 7th (Stata commands presented).

stset LPR_month, failure(LPR_event==1) scale(1)
sts graph, by(T_7th) risktable censored(single)
sts test T_7th

Figure: Kaplan-Meier estimates of progression-free survival of 51 patients with LA HSCC (Stata commands presented).

stset PFS_month, failure(PFS_event==1)
sts graph, risktable censored(single) plotopts(recast(line)) xlabel(0(12)36),48,60,72

Figure: Kaplan-Meier estimates of progression-free survival of T3 stage and T4 stage patients with LA HSCC 8th (Stata commands presented).

stset PFS_month, failure(PFS_event==1) scale(1)
sts graph, by(T_8th) risktable censored(single)
sts test T_8th

Figure: Kaplan-Meier estimates of progression-free survival of T3 stage and T4 stage patients with LA HSCC 7th (Stata commands presented).

stset PFS_month, failure(PFS_event==1) scale(1)
sts graph, by(T_7th) risktable censored(single)
sts test T_7th

Figure: Kaplan-Meier estimates of metastasis-free survival of 51 patients with LA HSCC (Stata commands presented).

stset MFS_month, failure(MFS_event==1)

sts graph, risktable censored(single) plotopts(recast(line)) xlabel(0(12)60)

Figure: Kaplan-Meier estimates of metastasis-free survival of T3 stage and T4 stage patients with LA HSCC 8th (Stata commands presented).

stset MFS_month, failure(MFS_event==1) scale(1)

sts graph, by(T_8th) risktable censored(single)

sts test T_8th

Figure: Kaplan-Meier estimates of metastasis-free survival of T3 stage and T4 stage patients with LA HSCC 7th (Stata commands presented).

stset MFS_month, failure(MFS_event==1) scale(1)

sts graph, by(T_7th) risktable censored(single)

sts test T_7th

Figure: Kaplan-Meier estimates of overall survival of 51 patients with LA HSCC (Stata commands presented).

stset OS_month, failure(OS_event==1)

sts graph, risktable censored(single) plotopts(recast(line)) xlabel(0(12)60)

Figure: Kaplan-Meier estimates of overall survival of T3 stage and T4 stage patients with LA HSCC 8th (Stata commands presented).

stset OS_month, failure(OS_event==1) scale(1)

sts graph, by(T_8th) risktable censored(single)

sts test T_8th

Figure: Kaplan-Meier estimates of overall survival of T3 stage and T4 stage patients with LA HSCC 7th (Stata commands presented).

stset OS_month, failure(OS_event==1) scale(1)

sts graph, by(T_7th) risktable censored(single)

sts test T_7th

Figure: treatment procedures and time frame of chemoimmunotherapy, radioimmunotherapy, and immunotherapy maintenance of 51 patients (Stata commands presented).

twoway (scatter ID Events)

Subgroups analyses

The difference of laryngeal preservation rate among III, IVa and IVb stages 8th (Stata commands presented).

stset LPR_month, failure(LPR_event==1) scale(1)
sts graph, by(Clinical_stages_8th) risktable censored(single)
sts test Clinical_stages_8th

The difference of laryngeal preservation rate among III, IVa and IVb stages 7th (Stata commands presented).

stset LPR_month, failure(LPR_event==1) scale(1)
sts graph, by(Clinical_stages_7th) risktable censored(single)
sts test Clinical_stages_7th

The difference of laryngeal preservation rate among N stages 8th (Stata commands presented).

stset LPR_month, failure(LPR_event==1) scale(1)
sts graph, by(N_8th) risktable censored(single)
sts test N_8th

The difference of laryngeal preservation rate among N stages 7th (Stata commands presented).

stset LPR_month, failure(LPR_event==1) scale(1)
sts graph, by(N_7th) risktable censored(single)
sts test N_7th

The difference in laryngeal preservation rate between T3 stage and T4 stage patients 8th (Stata commands presented).

stset LPR_month, failure(LPR_event==1) scale(1)
sts graph, by(T_8th) risktable censored(single)
sts test T_8th

The difference in laryngeal preservation rate between T3 stage and T4 stage patients 7th (Stata commands presented).

stset LPR_month, failure(LPR_event==1) scale(1)
sts graph, by(T_7th) risktable censored(single)
sts test T_7th

The difference of laryngeal preservation rate among the tumor sites of the pyriform sinus, postcricoid, and posterior hypopharyngeal wall (Stata commands presented).

stset LPR_month, failure(LPR_event==1) scale(1)
sts graph, by(Tumor_sites) risktable censored(single)
sts test Tumor_sites

The difference of progression-free survival rate among N stages 8th (Stata commands presented).

stset PFS_month, failure(PFS_event==1) scale(1)
sts graph, by(N_8th) risktable censored(single)
sts test N_8th

The difference of progression-free survival rate among N stages 7th (Stata commands presented).

stset PFS_month, failure(PFS_event==1) scale(1)
sts graph, by(N_7th) risktable censored(single)
sts test N_7th

The difference in progression-free survival rate between T3 stage and T4 stage patients 8th (Stata commands presented).

stset PFS_month, failure(PFS_event==1) scale(1)
sts graph, by(T_8th) risktable censored(single)
sts test T_8th

The difference in progression-free survival rate between T3 stage and T4 stage patients 7th (Stata commands presented).

stset PFS_month, failure(PFS_event==1) scale(1)
sts graph, by(T_7th) risktable censored(single)
sts test T_7th

The difference in progression-free survival rate was detected among III, IVa and IVb stages 8th (Stata commands presented).

stset PFS_month, failure(PFS_event==1) scale(1)
sts graph, by(Clinical_stages_8th) risktable censored(single)
sts test Clinical_stages_8th

The difference in progression-free survival rate was detected among III, IVa and IVb stages 7th (Stata commands presented).

stset PFS_month, failure(PFS_event==1) scale(1)
sts graph, by(Clinical_stages_7th) risktable censored(single)
sts test Clinical_stages_7th

The difference of progression-free survival rate among tumor sites of the pyriform sinus, postcricoid, and posterior hypopharyngeal wall (Stata commands presented).

stset PFS_month, failure(PFS_event==1) scale(1)
sts graph, by(Tumor_sites) risktable censored(single)
sts test Tumor_sites

The difference of metastasis-free survival rate among N stages 8th (Stata commands

presented).

stset MFS_month, failure(MFS_event==1) scale(1)

sts graph, by(N_8th) risktable censored(single)

sts test N_8th

The difference of metastasis-free survival rate among N stages 7th (Stata commands presented).

stset MFS_month, failure(MFS_event==1) scale(1)

sts graph, by(N_7th) risktable censored(single)

sts test N_7th

The difference of metastasis-free survival rate between T3 stage and T4 stage patients 8th (Stata commands presented).

stset MFS_month, failure(MFS_event==1) scale(1)

sts graph, by(T_8th) risktable censored(single)

sts test T_8th

The difference of metastasis-free survival rate between T3 stage and T4 stage patients 7th (Stata commands presented).

stset MFS_month, failure(MFS_event==1) scale(1)

sts graph, by(T_7th) risktable censored(single)

sts test T_7th

The difference of metastasis-free survival rate among III, IVa and IVb stages 8th (Stata commands presented).

stset MFS_month, failure(MFS_event==1) scale(1)

sts graph, by(Clinical_stages_8th) risktable censored(single)

sts test Clinical_stages_8th

The difference of metastasis-free survival rate among III, IVa and IVb stages 7th (Stata commands presented).

stset MFS_month, failure(MFS_event==1) scale(1)

sts graph, by(Clinical_stages_7th) risktable censored(single)

sts test Clinical_stages_7th

The difference of metastasis-free survival rate among tumor sites of the pyriform sinus, postcricoid, and posterior hypopharyngeal wall (Stata commands presented).

stset MFS_month, failure(MFS_event==1) scale(1)

sts graph, by(Tumor_sites) risktable censored(single)

sts test Tumor_sites

The difference of overall survival rate among N stages 8th (Stata commands presented).

stset OS_month, failure(OS_event==1) scale(1)

sts graph, by(N_8th) risktable censored(single)

sts test N_8th

The difference of overall survival rate among N stages 7th (Stata commands presented).

stset OS_month, failure(OS_event==1) scale(1)
sts graph, by(N_7th) risktable censored(single)
sts test N_7th

The difference of overall survival rate between T3 stage and T4 stage patients 8th (Stata commands presented).

stset OS_month, failure(OS_event==1) scale(1)
sts graph, by(T_8th) risktable censored(single)
sts test T_8th

The difference of overall survival rate between T3 stage and T4 stage patients 7th (Stata commands presented).

stset OS_month, failure(OS_event==1) scale(1)
sts graph, by(T_7th) risktable censored(single)
sts test T_7th

The difference of overall survival rate among III, IVa and IVb stages 8th (Stata commands presented).

stset OS_month, failure(OS_event==1) scale(1)
sts graph, by(Clinical_stages_8th) risktable censored(single)
sts test Clinical_stages_8th

The difference of overall survival rate among III, IVa and IVb stages 7th (Stata commands presented).

stset OS_month, failure(OS_event==1) scale(1)
sts graph, by(Clinical_stages_7th) risktable censored(single)
sts test Clinical_stages_7th

The difference of overall survival rate among tumor sites of the pyriform sinus, postcricoid, and posterior hypopharyngeal wall (Stata commands presented).

stset OS_month, failure(OS_event==1) scale(1)
sts graph, by(Tumor_sites) risktable censored(single)
sts test Tumor_sites

Estimated 1-year, 2-year and 3-year LPR rates (Stata commands presented).

ltable LPR_month LPR_event, survival failure intervals(12)

Estimated 1-year, 2-year and 3-year PFS rates (Stata commands presented).

ltable PFS_month PFS_event, survival failure intervals(12)

Estimated 1-year, 2-year and 3-year MFS rates (Stata commands presented).

ltable MFS_month MFS_event, survival failure intervals(12)

Rstimated 1-year, 2-year and 3-year OS rates (Stata commands presented).
ltable OS_month OS_event, survival failure intervals(12)

The ORR levels of tumor volume in group of patients with CPS < 1% CPS ≥ 1% (Stata commands presented).
tabstat VolumeResponse_detail, statistics(mean sd) by(PDL1_express) columns(variables)
anova VolumeResponse_detail PDL1_express

The ORR levels of tumor diameter in group of patients with CPS < 1% CPS ≥ 1% (Stata commands presented).
tabstat DiameterResponse_detail, statistics(mean sd) by(PDL1_express) columns(variables)
anova DiameterResponse_detail PDL1_express

The expression results of PD-L1 in the primary tumor site of patients with PR and SD patients (Stata commands presented).
tabulate Volume_evaluation PDL1_express, chi2 column row

Besides, post-hoc analysis about oncologic outcomes, tissue samples and blood samples of enrolled patients will be perform. The statistical analysis will be performed by Stata software and other methods.

Summary of changes – statistical analysis plan

Statistical analysis plan version: 1.1(2019-07-26)
Statistical analysis plan date: 2019-07-26

For Statistical analysis plan amendment # to:
Statistical analysis plan version: 1.2(2021-08-08)
Statistical analysis plan date: 2021-08-08

**#**	**Section**	**Change**	
		Version 1.1(2019-07-26)<br>Date (2019-07-26)	Version 1.2(2021-08-08)<br>Date (2021-08-08)
1	Study Design	cT3-4aN1-2M0 (AJCC 7th)	cT3-4aN0-2M0 (AJCC 7th)
2	Outcome	Primary outcome measure	Primary outcome measure (time frame: 9 weeks)
