# Supplementary material for: Camrelizumab-based induction chemoimmunotherapy in locally advanced stage hypopharyngeal carcinoma: phase II clinical trial
Source: Nat Commun. 2024 Jun 19;15:5251. doi: 10.1038/s41467-024-49121-3 (PMC11187213; doi:10.1038/s41467-024-49121-3)
Supplement: Supplementary file 2 — Description of Additional Supplementary Files [file 41467_2024_49121_MOESM2_ESM.pdf]

Supplementary Data 1.

Surgical treatments of 10 patients in this trial.

This table included ten patient ID, surgical treatment period, tumor involved sites of primary and cervical (CT+MRI+fibrolaryngoscope evaluation), presurgery cTNM (AJCC 8th), surgical treatments, postoperative pathologic evaluation, and postoperative pTNM (AJCC 8th).
