# Supplementary material for: Camrelizumab-based induction chemoimmunotherapy in locally advanced stage hypopharyngeal carcinoma: phase II clinical trial
Source: Nat Commun. 2024 Jun 19;15:5251. doi: 10.1038/s41467-024-49121-3 (PMC11187213; doi:10.1038/s41467-024-49121-3)
Supplement: Supplementary file 3 — Reporting Summary [file 41467_2024_49121_MOESM3_ESM.pdf]

nature portfolio

Corresponding author(s): Liang Zhou

Last updated by author(s): 2024.5.8

Reporting Summary

Nature Portfolio wishes to improve the reproducibility of the work that we publish. This form provides structure for consistency and transparency in reporting. For further information on Nature Portfolio policies, see our Editorial Policies and the Editorial Policy Checklist.

Statistics

For all statistical analyses, confirm that the following items are present in the figure legend, table legend, main text, or Methods section.

n/a	Confirmed	
☐	☒	The exact sample size (*n*) for each experimental group/condition, given as a discrete number and unit of measurement
☐	☒	A statement on whether measurements were taken from distinct samples or whether the same sample was measured repeatedly
☐	☒	The statistical test(s) used AND whether they are one- or two-sided<br>*Only common tests should be described solely by name; describe more complex techniques in the Methods section.*
☒	☐	A description of all covariates tested
☐	☒	A description of any assumptions or corrections, such as tests of normality and adjustment for multiple comparisons
☐	☒	A full description of the statistical parameters including central tendency (e.g. means) or other basic estimates (e.g. regression coefficient) AND variation (e.g. standard deviation) or associated estimates of uncertainty (e.g. confidence intervals)
☐	☒	For null hypothesis testing, the test statistic (e.g. *F*, *t*, *r*) with confidence intervals, effect sizes, degrees of freedom and *P* value noted<br>*Give P values as exact values whenever suitable.*
☒	☐	For Bayesian analysis, information on the choice of priors and Markov chain Monte Carlo settings
☒	☐	For hierarchical and complex designs, identification of the appropriate level for tests and full reporting of outcomes
☒	☐	Estimates of effect sizes (e.g. Cohen's *d*, Pearson's *r*), indicating how they were calculated

Our web collection on statistics for biologists contains articles on many of the points above.

Software and code

Policy information about availability of computer code

Data collection	Data collection was performed by Microsoft Office EXCEL 2019 and LSRFortessa (BD Biosciences) in this study.
Data analysis	Data was analyzed by Stata (version 17.0) and GraphPad Prism project (version 9.4.1), and FlowJo software (version 10.0) in this study.

For manuscripts utilizing custom algorithms or software that are central to the research but not yet described in published literature, software must be made available to editors and reviewers. We strongly encourage code deposition in a community repository (e.g. GitHub). See the Nature Portfolio guidelines for submitting code & software for further information.

Data

Policy information about availability of data

All manuscripts must include a data availability statement. This statement should provide the following information, where applicable:

- Accession codes, unique identifiers, or web links for publicly available datasets
- A description of any restrictions on data availability
- For clinical datasets or third party data, please ensure that the statement adheres to our policy

The protocol of this study is available as Supplementary Note 1 in the Supplementary Information file. The clinical characteristics, oncologic outcome, adverse events, radiographic, pathological, and exploratory analysis data of this study underlying the results are available in the Supplementary Information file. The raw sequence data of WES DNA reported in this paper have been deposited in the Genome Sequence Archive (Genomics, Proteomics & Bioinformatics 2021) in National Genomics Data Center (Nucleic Acids Res 2022), China National Center for Bioinformation / Beijing Institute of Genomics, Chinese Academy of Sciences (GSA-Human: HRA006987) that are publicly accessible at https://ngdc.cncb.ac.cn/gsa-human. Access to the WES DNA data can be requested for research purpose by

completing the application form GSA for Human System and is granted by the Data Access Committee, and the guidance can be found at the website. The de-identified participant data, including radiographic images and blood test results of patients in the current study are available from the corresponding author (Liang Zhou zhoulent@126.com) for research purpose according to policy of Ethics Committees of Eye & ENT Hospital, Fudan University, after completing the trial for five years. The key points of the Committee policy are for scientific human cancer research and are not harmful to people's health. When the access is granted, the data will be available to the requester. The remaining data of this study are available in the Article, Supplementary Information or Source Data file. Source data are provided with this paper.

Research involving human participants, their data, or biological material

Policy information about studies with human participants or human data. See also policy information about sex, gender (identity/presentation), and sexual orientation and race, ethnicity and racism.

Reporting on sex and gender	Patients were collected regardless of sex or gender, and no sex- and gender-based analysis.
Reporting on race, ethnicity, or other socially relevant groupings	No race, ethnicity, or other socially relevant groupings in this study.
Population characteristics	Population characteristics of 51 patients were enrolled and displayed in Table 1 and Supplementary table 1 in this study.
Recruitment	This open-label, single-arm, phase II, prospective, and single-center study was performed at Eye & ENT Hospital, Fudan University. Eligibly patients were with curable purpose of local therapies (cT3-4aN0-2M0); had to be treated with total laryngectomy; aged 18 to 70 years old; did not received any anti-tumor therapy previously; an Eastern Cooperative Oncology Group performance performance status of 0 - 2; an estimated life expectancy of six months or more; and adequate system organ function. All patients were enrolled according to inclusion and exclusion criteria (ClinicalTrials.gov, No. NCT04156698). Each patient provided signed informed consent before participating in this trial. There is no potential self-selection bias and other biases.
Ethics oversight	This trial was performed in accordance with the Declaration of Helsinki and the International Conference on Good Clinical Practice guidelines. The Ethics Committees of Eye & ENT Hospital, Fudan University, approved the trial protocol and treatments. All patients provided written informed consent before enrollment.

Note that full information on the approval of the study protocol must also be provided in the manuscript.

Field-specific reporting

Please select the one below that is the best fit for your research. If you are not sure, read the appropriate sections before making your selection.

- [x] Life sciences
- [ ] Behavioural & social sciences
- [ ] Ecological, evolutionary & environmental sciences

For a reference copy of the document with all sections, see nature.com/documents/nr-reporting-summary-flat.pdf

Life sciences study design

All studies must disclose on these points even when the disclosure is negative.

Sample size	The sample size was calculated based on the primary endpoint of ORR, which was predicted to be 80% in this study, and the history control value was 60% from our previous data, with 80% detection power and using a two-sided at the formal statistical boundary for the significance of 0.05. Forty- three cases were required to assess the antitumor activity of camrelizumab, plus 15% of cases due to loss to follow-up; overall, 51 participants were required to enroll in this trial.
Data exclusions	Two patients were excluded: 1 patient withdrew consent and 1 patient did not meet inclusion criteria, and finally 51 patients were enrolled and analyzed in this study (Fig. 1 of manuscript).
Replication	Not applicable. It was an open-label, single-arm, single-center, phase II, prospective, and non-randomized clinical study. No preclinical data that could be replicated were provided in this study. DNA sequence, immunohistochemistry, and whole peripheral blood Flow cytometer were performed in this study. However, due to limited tissue and blood samples, we only performed these tests once.
Randomization	Not applicable. It was an open-label, single-arm, single-center, phase II, prospective, and non-randomized clinical study.
Blinding	Not applicable. This was an open-label, single-arm, single-center, phase II, prospective, and non-randomized clinical study. Patients were enrolled in one consecutive cohort, the blinding method did not apply.

Reporting for specific materials, systems and methods

We require information from authors about some types of materials, experimental systems and methods used in many studies. Here, indicate whether each material, system or method listed is relevant to your study. If you are not sure if a list item applies to your research, read the appropriate section before selecting a response.

Materials & experimental systems

n/a	Involved in the study
☐	☒ Antibodies
☒	☐ Eukaryotic cell lines
☒	☐ Palaeontology and archaeology
☒	☐ Animals and other organisms
☐	☒ Clinical data
☒	☐ Dual use research of concern
☒	☐ Plants

Methods

n/a	Involved in the study
☒	☐ ChIP-seq
☐	☒ Flow cytometry
☒	☐ MRI-based neuroimaging

Antibodies

Antibodies used	Mouse Anti-Human PD-L1 (DAKO Cat#M3653 22C3), used at 1:50 dilution.<br>PerCP/Cyanine5.5 anti-human CD3 (Biolegend Cat#317336 OKT3) , used at 5 μL/test.<br>PE/Cyanine7 anti-human CD19 (Biolegend Cat#302216 HIB19), used at 5 μL/test.<br>APC/Fire™ 750 anti-human CD4 (Biolegend Cat#300560 RPA-Ta), used at 5 μL/test.<br>FITC anti-human CD8a (Biolegend Cat#300906 HIT8a), used at 5 μL/test.<br>Brilliant Violet 421™ anti-human CD123 (BioLegend Cat#306018 6H6), used at 5 μL/test.<br>FITC anti-human CD303 (BioLegend Cat#354208 201A), used at 5 μL/test.<br>Brilliant Violet 711™ anti-human CD45 (BioLegend Cat#304050 HI30), used at 5 μL/test.<br>PE/Cyanine7 anti-human CD11c (BioLegend Cat#301608 3.9), used at 5 μL/test.
Validation	All of the antibodies used in this study were validated for IHC and FACS use in human specimens by the manufacturers. The detailed antibody information and antibody validation procedures were described on the following respective manufacturers' websites: https://www.agilent.com.cn/en/dako-products; https://www.biolegend.com/en-gb.

Clinical data

Policy information about clinical studies

All manuscripts should comply with the ICMJEguidelines for publication of clinical research and a completedCONSORT checklist must be included with all submissions.

Clinical trial registration	This trial was registered with ClinicalTrials.gov, No. NCT04156698.
Study protocol	Study protocol of this study was submitted as Supplementary Note1 in Supplementary information.
Data collection	This trial was an open-label, single-arm, phase II, prospective, and single-center study, and was performed at Eye & ENT Hospital, Fudan University. Between May 21, 2020, and November 15, 2023, 53 patients were screened, and 51 were enrolled and analyzed in this study. Patients were aged from 35 to 69 years old, and were confirmed to be locally advanced hypopharyngeal squamous cell carcinoma. The authorization of the collection and release of the data from the Ethics Committees of Eye & ENT Hospital were obtained.
Outcomes	The primary endpoint was objective response rate (ORR) evaluated by Response Evaluation Criteria in Solid Tumors v.1.1. Fifty-one patients were enrolled, and the median duration of follow-up was 23.7 months. After induction therapy, the ORR was 82.4% (42/51). The most common AE was alopecia (100%), followed by reactive cutaneous capillary endothelial proliferation (90.2%) in the induction therapy period. Twenty-six (51.0%) patients experienced grade three or worse AEs. As the three-year outcomes are immature and no distant metastatic event has occurred so far, we reported three outcomes except MFS at one and two years. The one-year overall survival, progression-free survival, and laryngeal function preservation rates were 93.7%, 85.3%, and 75.0%, respectively. The two-year overall survival, progression-free survival, and laryngeal function preservation rates were 83.0%, 77.1%, and 70.0%, respectively. Patients with partial response showed higher tumor mutational burden and CD8+ in CD3+CD19- total T lymphocytes than those of patients with stable disease.

Plants

Seed stocks	None
Novel plant genotypes	None
Authentication	None

Flow Cytometry

Plots

Confirm that:

- [x] The axis labels state the marker and fluorochrome used (e.g. CD4-FITC).
- [x] The axis scales are clearly visible. Include numbers along axes only for bottom left plot of group (a 'group' is an analysis of identical markers).
- [x] All plots are contour plots with outliers or pseudocolor plots.
- [x] A numerical value for number of cells or percentage (with statistics) is provided.

Methodology

Sample preparation	Whole peripheral blood samples were centrifuged at 3000 rpm for 10 minutes to remove plasma, and the remaining blood cells were lysed with RBC lysis buffer (4300, eBioscience) at room temperature for five minutes. Cells suspension was washed in staining buffer and stained with fluorochrome-conjugated monoclonal antibodies for cell surface markers to identify different lymphocytes and their subgroups. Zombie UV Fixable Viability kit (423107, BioLegend) was used to assess live status. Cells were first incubated with the live/dead dye for 20 min at room temperature, then were washed and stained for 30 min at room temperature in the dark for cell surface markers to attach.
Instrument	Data were acquired by using an LSRFortessa (BD Biosciences)
Software	Data were analyzed by using FlowJo software (version 10.0).
Cell population abundance	In PBMC: Live cell was ~98% of single cell population; CD45+ was ~ 90% of live cell; CD19+ was ~ 5%-15% of CD45+ cell; CD3+ was ~ 60 - 80% of CD45+ cell; CD123+ CD303+ was ~ 0.1-2% of CD11c- cell.
Gating strategy	CD3+ and CD19+ were both gated in CD45+, CD45+ was gated in live cells (from single cells).<br>CD4+ and CD8+ were both gated in CD3+ T lymphocytes (from single cells).<br>CD123+ CD303+ pDC was gating in CD45+CD11c- live cells (single cells).

- [x] Tick this box to confirm that a figure exemplifying the gating strategy is provided in the Supplementary Information.
